# Correction: Rational Development of an Attenuated Recombinant Cyprinid Herpesvirus 3 Vaccine Using Prokaryotic Mutagenesis and In Vivo Bioluminescent Imaging

**DOI:** 10.1371/journal.ppat.1004861

**Published:** 2015-04-22

**Authors:** The PLOS Pathogens Staff

## Notice of Republication

This article was republished on April 6, 2015, to correct errors that were introduced during the typesetting process. Fig 4 was incorrectly cropped, and we have also corrected some typographical errors in the text. The publisher apologizes for these errors. Please download this article again to view the correct version. The originally published, uncorrected article and the republished, corrected article are provided here for reference.

## Supporting Information

S1 File Originally published, uncorrected article. (PDF)

S2 File Republished, corrected article. (PDF)
