# Supplementary figures and images for: Rational Development of an Attenuated Recombinant Cyprinid Herpesvirus 3 Vaccine Using Prokaryotic Mutagenesis and In Vivo Bioluminescent Imaging
Source: PLoS Pathog. 2015 Feb 20;11(2):e1004690. doi: 10.1371/journal.ppat.1004690 (PMC4336323; doi:10.1371/journal.ppat.1004690)

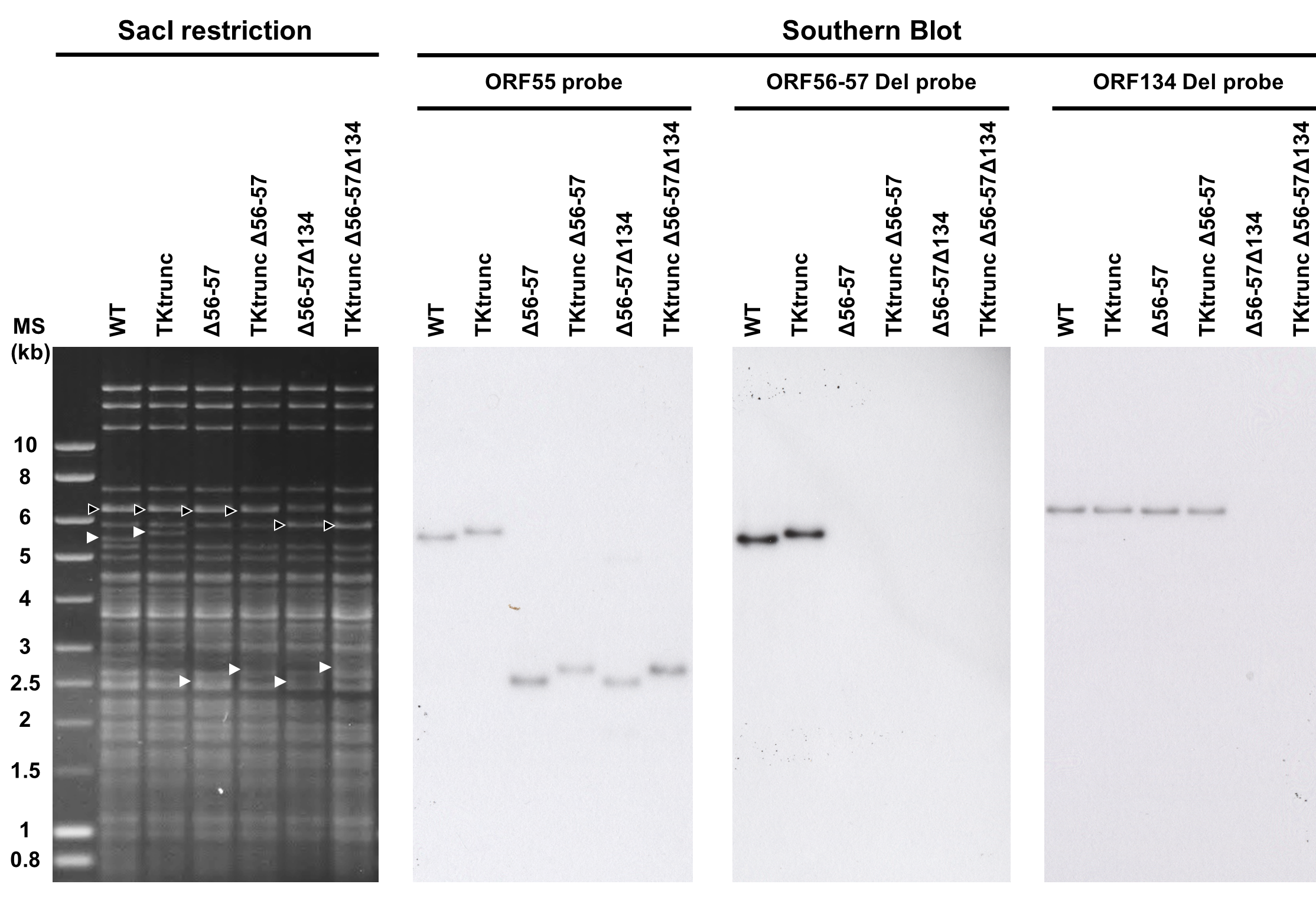

Supplement: S1 Fig — The indicated strains were analyzed by SacI restriction (left) and Southern blotting using ORF55, ORF56-57 Del, and ORF134 Del probes. Black and white arrowheads indicate fragments containing ORF134 and ORF56-57, respectively. Markers sizes (MS) are indicated on the left. (TIF) [file ppat.1004690.s001.tif]

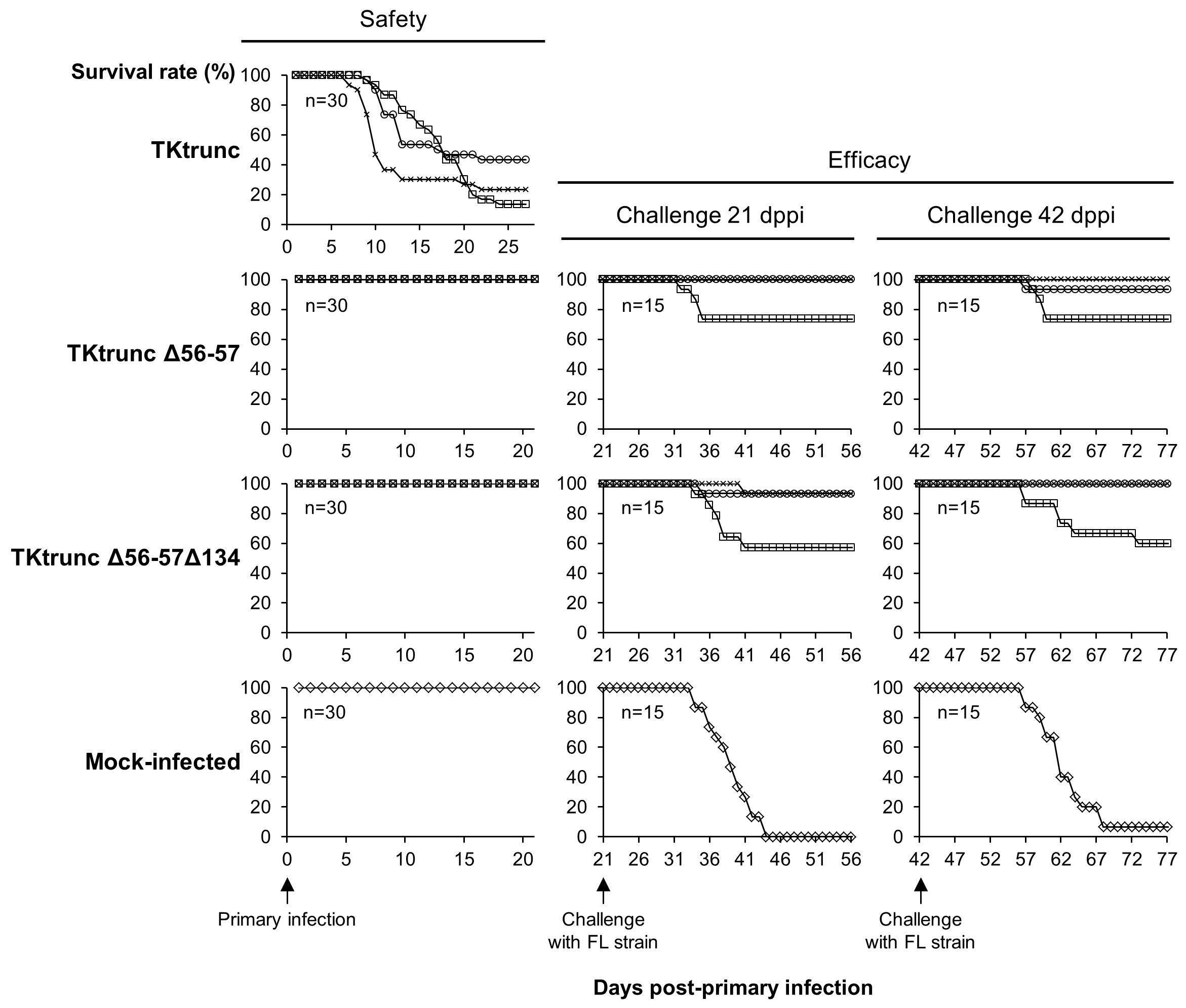

Supplement: S2 Fig — The safety and efficacy of the indicated recombinant strains was tested in common carp (average weight 4.41 g ± 1.78 g, 7 months old). On day 0, fish were infected for 2 h by immersion in water containing 4 (□), 40 (○), or 400 (x) pfu/ml. Safety was investigated by measuring the survival rate for 21 days in a group of 30 carp. Efficacy was tested at 21 and 42 dppi. Mock-infected fish and fish that survived the primary infection were distributed in tanks (n = 15) and challenged by cohabitation with fish infected with the FL strain. (TIF) [file ppat.1004690.s002.tif]

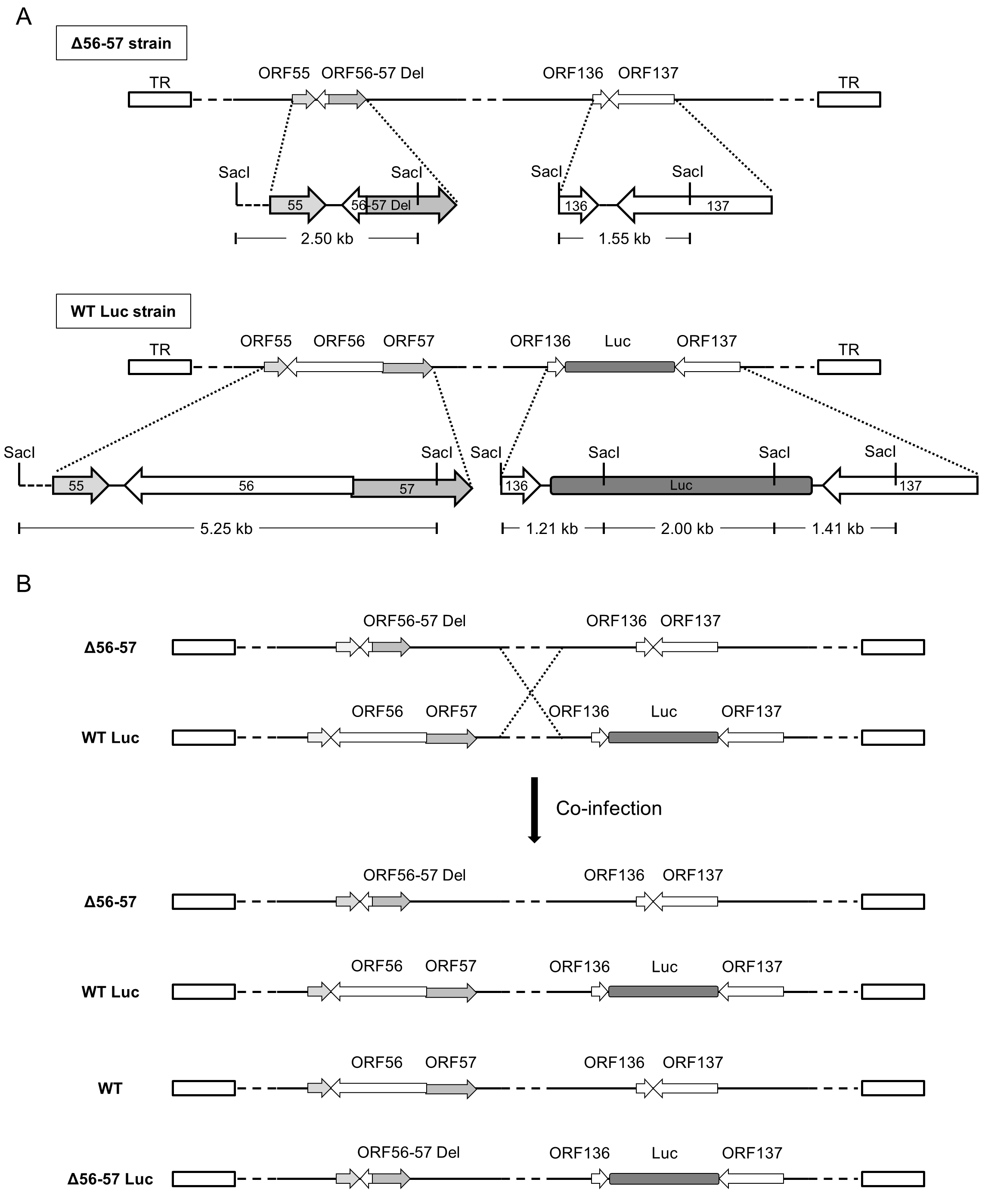

Supplement: S3 Fig — (A) Molecular structure of the Δ56-57 strain and WT Luc strain used as parental strains for production of the Δ56-57 Luc strain by co-infection in eukaryotic cells. ORFs are represented by white or grey arrows, the Luc cassette by a grey rectangle, and CyHV-3 terminal repeats by white rectangles. The positions of SacI restriction sites and restriction fragment lengths are shown below each genotype. (B) Schematic representation of the method used to produce recombinant strains by co-infection of two parental strains. The molecular structures of the four possible recombinant strains resulting from unique or multiple cross-overs are illustrated. (TIF) [file ppat.1004690.s003.tif]

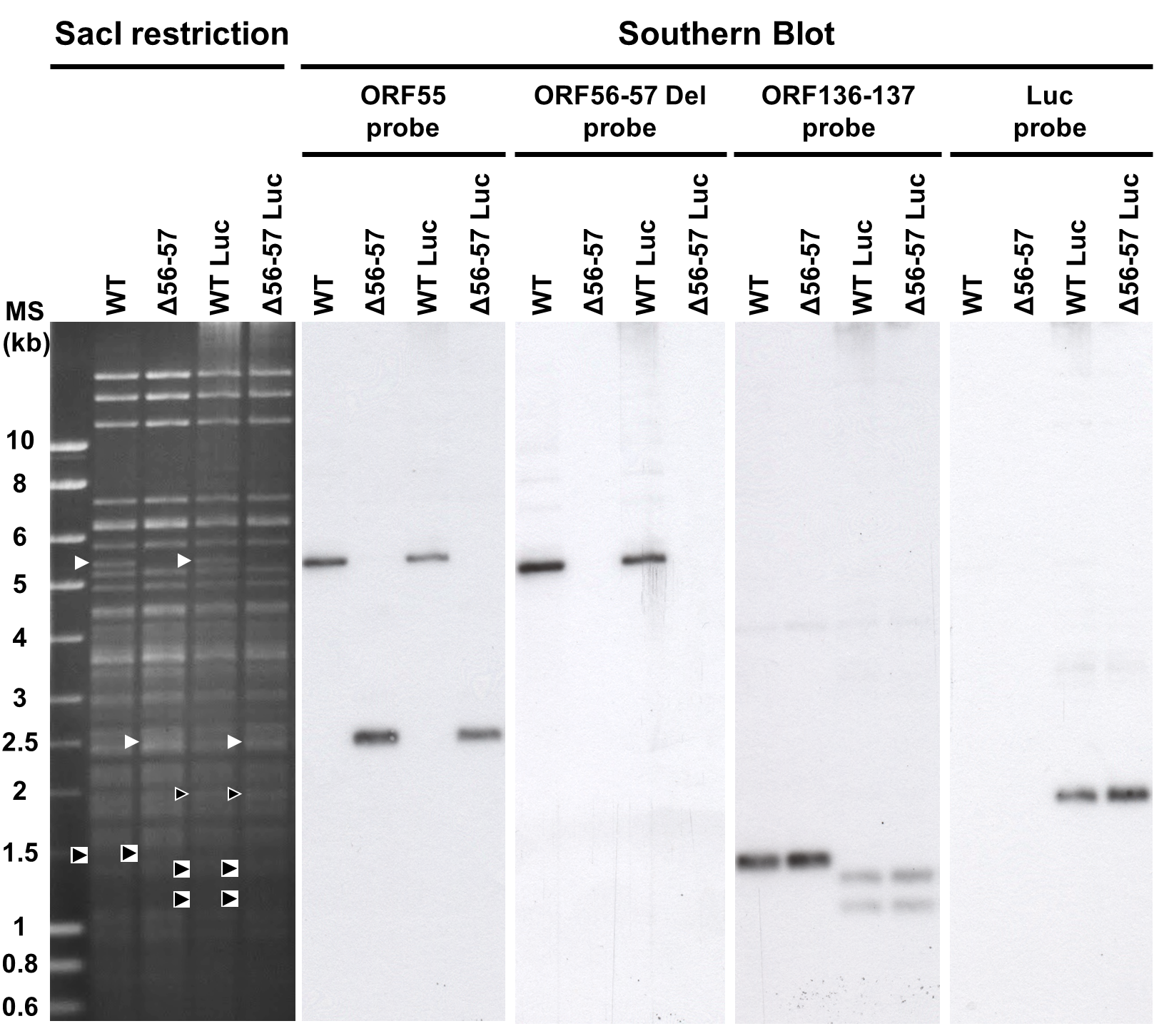

Supplement: S4 Fig — The indicated strains were analyzed by SacI restriction (left) and Southern blotting using ORF55, ORF56-57 Del, ORF136-137, and Luc probes. White and white-outlined black arrowheads indicate fragments containing ORF56-57 loci and ORF136-137 loci, respectively. Black arrowheads indicate the restriction fragment containing most of the Luc cassette sequence. Marker sizes (MS) are indicated on the left. (TIF) [file ppat.1004690.s004.tif]

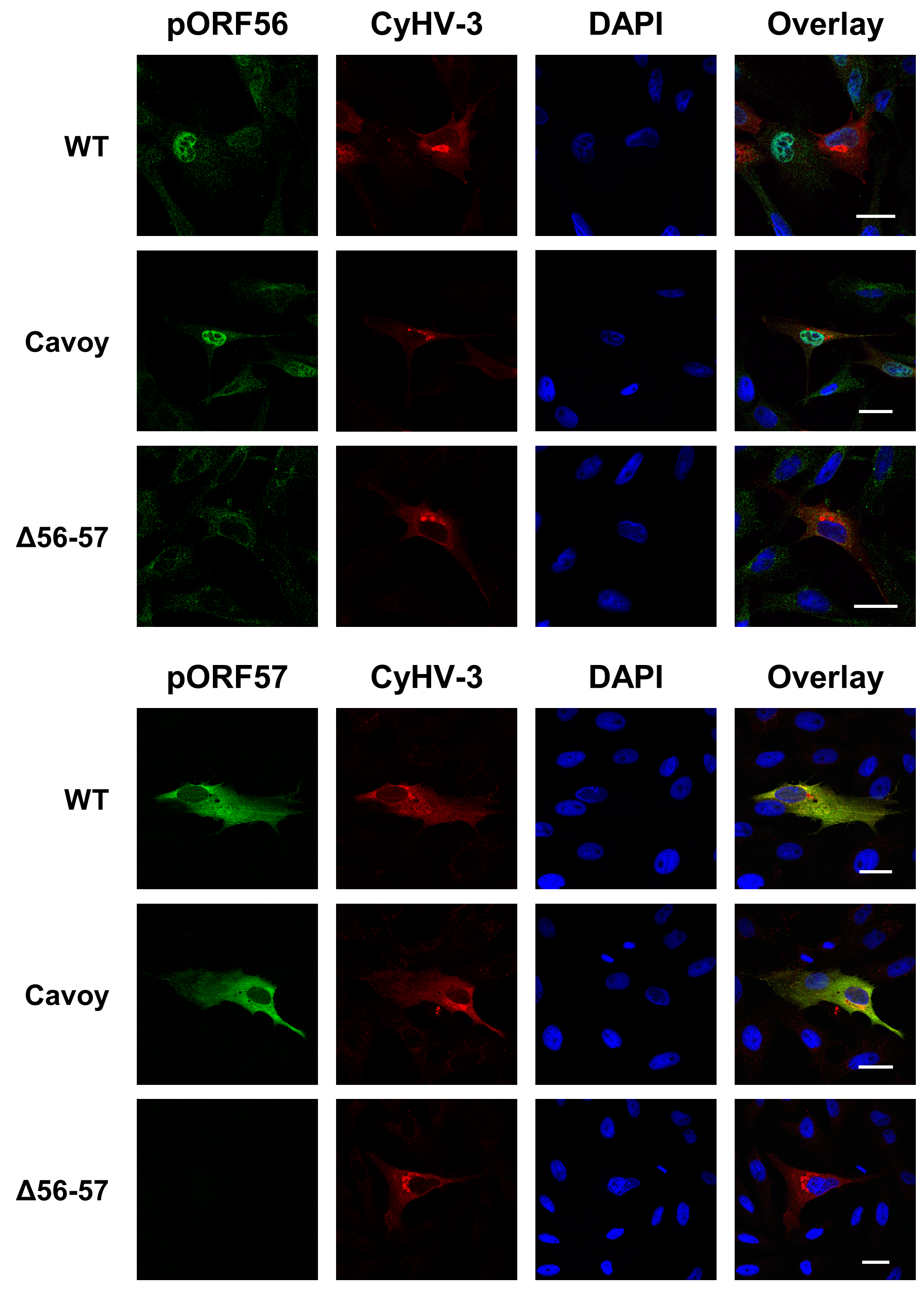

Supplement: S5 Fig — CCB cells were infected with the indicated strains. One day post-infection, cells were treated for indirect immunofluorescent staining and confocal microscopic analysis of pORF56 or pORF57 (green), CyHV-3 structural proteins (red), and cell nuclei (blue). The overlay represents the superposition of the three channels. White scale bars = 20 μm. (TIF) [file ppat.1004690.s005.tif]
