# Supplementary material for: Correction: Rational Development of an Attenuated Recombinant Cyprinid Herpesvirus 3 Vaccine Using Prokaryotic Mutagenesis and In Vivo Bioluminescent Imaging
Source: PLoS Pathog. 2015 Apr 22;11(4):e1004861. doi: 10.1371/journal.ppat.1004861 (PMC4406827; doi:10.1371/journal.ppat.1004861)
Supplement: S1 File — (PDF) [file ppat.1004861.s001.pdf]

PLOS | PATHOGENS

RESEARCH ARTICLE

Rational Development of an Attenuated Recombinant Cyprinid Herpesvirus 3 Vaccine Using Prokaryotic Mutagenesis and In Vivo Bioluminescent Imaging

Maxime Boutier[1], Maygane Ronsmans[1], Ping Ouyang[1¤], Guillaume Fournier[1], Anca Reschner[1], Krzysztof Rakus[1], Gavin S. Wilkie[2], Frédéric Farnir[3], Calixte Bayrou[4], François Lieffrig[5], Hong Li[6], Daniel Desmecht[4], Andrew J. Davison[2], Alain Vanderplasschen[1]*

1 Immunology-Vaccinology, Department of Infectious and Parasitic Diseases, Fundamental and Applied Research for Animals & Health (FARAH), Faculty of Veterinary Medicine, University of Liège, Liège, Belgium, **2** MRC—University of Glasgow Centre for Virus Research, Glasgow, United Kingdom, **3** Biostatistics and Bioinformatics, Fundamental and Applied Research for Animals & Health (FARAH), Faculty of Veterinary Medicine, University of Liège, Liège, Belgium, **4** Pathology, Department of Morphology and Pathology, Fundamental and Applied Research for Animals & Health (FARAH), Faculty of Veterinary Medicine, University of Liège, Liège, Belgium, **5** Fish Pathology Lab, Department of Biotechnology, CER Groupe, Marloie, Belgium, **6** USDA-ARS-ADRU, Washington State University, Pullman, Pullman, Washington, United States of America

¤ Current address: Key Laboratory of Animal Disease, College of Veterinary Medicine, Sichuan Agricultural University, Chengdu, Sichuan, China
* A.vdplasschen@ulg.ac.be

CrossMark
click for updates

OPEN ACCESS

Citation: Boutier M, Ronsmans M, Ouyang P, Fournier G, Reschner A, Rakus K, et al. (2015) Rational Development of an Attenuated Recombinant Cyprinid Herpesvirus 3 Vaccine Using Prokaryotic Mutagenesis and In Vivo Bioluminescent Imaging. PLoS Pathog 11(2): e1004690. doi:10.1371/journal.ppat.1004690

Editor: Lindsey Hutt-Fletcher, Louisiana State University Health Sciences Center, United States

Received: November 29, 2014

Accepted: January 20, 2015

Published: February 20, 2015

Copyright: This is an open access article, free of all copyright, and may be freely reproduced, distributed, transmitted, modified, built upon, or otherwise used by anyone for any lawful purpose. The work is made available under the Creative Commons CC0 public domain dedication.

Data Availability Statement: All relevant data are within the paper and its Supporting Information files.

Funding: This work was supported by MSD Animal Health grant KHV2013-2014 to AV, by Belgian Science Policy grant R.SSTC.0454-BELVIR IAP7/45 to AV, by FNRS grants R.FNRS.2165 2.4622.10 and R.FNRS.2597-1.A290.12 to AV and by UK Medical Research Council grant MC_UU_12014/3 to AD. The funders had no role in study design, data collection and analysis, decision to publish, or preparation of the manuscript.

Abstract

Cyprinid herpesvirus 3 (CyHV 3) is causing severe economic losses worldwide in common and koi carp industries, and a safe and efficacious attenuated vaccine compatible with mass vaccination is needed. We produced single deleted recombinants using prokaryotic mutagenesis. When producing a recombinant lacking open reading frame 134 (ORF134), we unexpectedly obtained a clone with additional deletion of ORF56 and ORF57. This triple deleted recombinant replicated efficiently in vitro and expressed an in vivo safety/efficacy profile compatible with use as an attenuated vaccine. To determine the role of the double ORF56-57 deletion in the phenotype and to improve further the quality of the vaccine candidate, a series of deleted recombinants was produced and tested in vivo. These experiments led to the selection of a double deleted recombinant lacking ORF56 and ORF57 as a vaccine candidate. The safety and efficacy of this strain were studied using an in vivo bioluminescent imaging system (IVIS), qPCR, and histopathological examination, which demonstrated that it enters fish via skin infection similar to the wild type strain. However, compared to the parental wild type strain, the vaccine candidate replicated at lower levels and spread less efficiently to secondary sites of infection. Transmission experiments allowing water contamination with or without additional physical contact between fish demonstrated that the vaccine candidate has a reduced ability to spread from vaccinated fish to naïve sentinel cohabitants. Finally, IVIS analyses demonstrated that the vaccine candidate

Competing Interests: The present study was financially supported at least in part by MSD animal health. This does not alter our adherence to all PLOS policies on sharing data and materials.

induces a protective mucosal immune response at the portal of entry. Thus, the present study is the first to report the rational development of a recombinant attenuated vaccine against CyHV 3 for mass vaccination of carp. We also demonstrated the relevance of the CyHV 3 carp model for studying alloherpesvirus transmission and mucosal immunity in teleost skin.

Author Summary

Common carp, and its colorful ornamental variety koi, is one of the most economically valuable species in aquaculture. Since the late 1990s, the common and koi carp culture industries have suffered devastating worldwide losses due to cyprinid herpesvirus 3 (CyHV-3). In the present study, we report the development of an attenuated recombinant vaccine against CyHV-3. Two genes were deleted from the viral genome, leading to a recombinant virus that is no longer capable of causing the disease but can be propagated in cell culture (for vaccine production) and infect fish when added to the water, thereby immunizing the fish. This attenuated recombinant vaccine also had a drastic defect in spreading from vaccinated to non-vaccinated cohabitant fish. The vaccine induced a protective mucosal immune response capable of preventing the entry of virulent CyHV-3 and is compatible with the simultaneous vaccination of a large number of carp by simply immersing the fish in water containing the vaccine. This vaccine represents a promising tool for controlling the most dreadful disease ever encountered by the carp culture industries. In addition, the present study highlights the importance of the CyHV-3 - carp model for studying alloherpesvirus transmission and mucosal immunity in teleost skin.

Introduction

Aquaculture is currently one of the world's fastest growing food production sectors, with an annual growth rate of 6.2% between 2000 and 2012 [1]. Global aquaculture currently provides half of the fish consumed worldwide. However, aquaculture is suffering important economic losses due to outbreaks of infectious and parasitic diseases [2–4], which are promoted by high rearing densities under artificial conditions [5], the efficient abiotic vector properties of water [5], and the international trade of genitors and fingerlings [6].

Inland aquaculture of freshwater finfishes dominates global aquaculture, representing 57.9% (38.6 million tons) of global production of aquaculture, far greater than mollusks (22.8%), crustaceans (9.7%), and mariculture of finfishes (8.3%) and other aquatic animals (1.3%) [1]. The common carp (*Cyprinus carpio*) is one of the oldest cultivated freshwater fish species. In China, carp cultivation dates back to at least the 5th century BC, and in Europe carp farming began during the Roman Empire [7]. Common carp is currently one of the most economically valuable species in aquaculture; it is one of the main fish species cultivated for human consumption, with worldwide production of 3.8 million tons (the third most important species based on the number of tons produced) in 2012, representing 9.8% of all freshwater fish production and US$5.2 billion [8]. Common carp is also produced and stocked in fishing areas for angling purposes. In addition, its colorful ornamental varieties (koi carp) grown for personal pleasure and competitive exhibitions represent one the most expensive markets for individual freshwater fish, with some prize-winners being sold for US$10,000-1,000,000 [9].

Koi herpesvirus (KHV), also known as cyprinid herpesvirus 3 (CyHV-3; species *Cyprinid herpesvirus 3*, genus *Cyprinivirus*, family *Alloherpesviridae*, order *Herpesvirales*), is the etiological agent of an emerging and mortal disease in common and koi carp [10]. Since its emergence in the late 1990s, this highly contagious disease has caused severe economic losses worldwide in both common and koi carp culture industries [9,11,12]. Outbreaks of CyHV-3 disease are associated with a mortality rate of 80–100% [9]. As an example, outbreaks of CyHV-3 in Indonesia in 2002 and 2003 caused an estimated economic loss of US$15 million [13,14].

The economic losses caused by infectious and parasitic diseases in aquaculture have motivated the development of efficient prophylactic vaccines [2,3]. In addition to the safety/efficacy issues that apply to all vaccines independent of the target species (humans or animals), vaccines for fish and production animals in general are under additional constraints. First, the vaccine must be compatible with mass vaccination and administered via a single dose as early as possible in life. Second, the cost-benefit ratio should be as low as possible, implying the lowest cost for vaccine production and administration [5]. Ideally, cost-effective mass vaccination of young fish is performed by bath vaccination, meaning that the fish are immersed in water containing the vaccine [15]. This procedure allows vaccination of a large number of subjects when their individual value is still low and their susceptibility to the disease highest [15]. The use of injectable vaccines for mass vaccination of fish is restricted to limited circumstances, i.e. when the value of an individual subject is relatively high and when vaccination can be delayed until an age when the size of the fish is compatible with their manipulation [16,17].

Various anti-CyHV-3 vaccine candidates have been developed [18–27]. Injectable DNA vaccines are efficacious under experimental conditions [21,22] but incompatible with most of the field constraints described above (i.e., the value of an individual common carp is very low and they should be vaccinated when only a few grams). In contrast, attenuated vaccines could meet these constraints but raise safety concerns, such as residual virulence, reversion to virulence, and spreading from vaccinated to naïve subjects. A conventional anti-CyHV-3 attenuated vaccine has been developed by serial passages in cell culture and UV irradiation [18–20,26,27]. This vaccine is commercialized in Israel by the KoVax Company for the vaccination of koi and common carp by immersion in water containing the attenuated strain and was recently launched in the US market under the name Cavoy but was withdrawn from sale after just a year. This vaccine has two major disadvantages. First, the attenuated strain has residual virulence for fish weighing less than 50 g [18,27]. Second, the determinism of the attenuation is unknown, and consequently, reversions to a pathogenic phenotype cannot be excluded [28].

Due to scientific advances in molecular biology and molecular virology, the development of attenuated vaccines is evolving from empirical to rational design [28–30]. A viral genome can be edited to delete genes encoding virulence factors in such a way that reversion to virulence can be excluded. This approach has been tested for CyHV-3 by targeting different genes thought to encode virulence factors, such as open reading frame (ORF) 16, ORF55, ORF123, and ORF134, which encode a G protein-coupled receptor, thymidine kinase (TK), deoxyuridine triphosphatase, and an Interleukine-10 (IL-10) homolog, respectively. Unfortunately, none of the recombinants lacking these genes express a safety/efficacy profile compatible with use as an attenuated recombinant vaccine [23,24,31].

In the present study, we took advantage of a recombinant strain that was obtained by accident. This strain was deleted of three genes and expressed a safety/efficacy profile compatible with use as an attenuated vaccine. Using prokaryotic recombination technologies, a series of recombinants was produced to identify the determinism of the attenuation and to improve further some properties of the vaccine candidate. Based on the results, a double deleted strain was selected as a vaccine candidate. The safety and efficacy of this strain as a recombinant

attenuated vaccine against CyHV-3 were investigated using various approaches, including an in vivo bioluminescent imaging system (IVIS). Taken together, the results of the present study demonstrate that this vaccine candidate is appropriate for safe and efficacious mass vaccination of carp against CyHV-3, inducing a protective mucosal immune response at the portal of entry. In addition to its importance for applied research, the present study is also important for fundamental research by demonstrating the potential of the CyHV-3 - carp model for studying the transmission of members of the familly *Alloherpesviridae* and mucosal immunity in teleost skin.

Results

Determinism of the attenuated phenotype observed for a triple deleted CyHV-3 strain

In an earlier study, we produced a CyHV-3 recombinant strain lacking ORF134, which encodes a viral IL-10 [31]. This recombinant was produced using a bacterial artificial chromosome (BAC) clone of the CyHV-3 FL strain and prokaryotic recombination technologies. To reconstitute infectious viral particles encoding a wild-type ORF55 locus (encoding TK) in which the BAC cassette was inserted, the FL BAC ORF134 Del galactokinase (*galK)* plasmid (deleted for ORF134) was co-transfected with the pGEMT-TK plasmid in permissive *Cyprinus carpio* brain (CCB) cells. For one of the viral clones we obtained, this procedure led to unexpected non-homologous recombination between the pGEMT vector and the beginning of ORF57, leading to reversion to a wild-type ORF55 locus and deletion of CyHV-3 genome from coordinates 97001 to 99726. This deleted region encodes most of ORF56 and the beginning of ORF57 (Fig. 1A). Despite this triple deletion (ORF56-57 and ORF134), this strain replicated efficiently in vitro, reaching a titer of 10^6 plaque forming units (pfu)/ml. Moreover, it expressed in vivo a safety/efficacy profile compatible with use as an attenuated vaccine for vaccination of carp against CyHV-3 by immersion in water containing the virus.

Here, we produced and tested a series of recombinant strains to establish the contribution of the ORF134 and the ORF56-57 deletions to the observed safety/efficacy profile of the triple deleted recombinant described above. Based on our recent study demonstrating that ORF134 is not essential for virulence in vivo [31], we hypothesized that the attenuated phenotype of the triple deleted strain was mainly, if not exclusively, determined by the ORF56-57 deletion. However, deletion of ORF134, which encodes an IL-10 homolog, could potentially contribute to the safety observed for the triple recombinant and/or the immune response it induced. To test these hypotheses, two groups of recombinants were produced independently using BAC cloning technologies according to the strategy described in Fig. 1B. Both groups encoded the ORF56-57 deletion (Fig. 1A) encompassing coordinates 97001 to 99750. This deletion was slightly longer on the 3' end than the deletion observed in the triple deleted strain described above in order to remove two potential alternative ATG start codons present in the beginning of ORF57. In addition to the ORF56-57 deletion, one of the groups also encoded an ORF134 deletion. When reconstituting infectious virions from recombinant BAC plasmids, the BAC cassette was removed by homologous recombination, leading to a wild-type ORF55 locus, or by cre-loxP-mediated excision, leading to a truncated ORF55 locus (TKtrunc genotype). The molecular structures of all recombinant strains were controlled by SacI restriction fragment length polymorphism (RFLP), Southern blot analysis (S1 Fig.), and sequencing of the genome region encoding ORF55 to ORF57. Strains encoding wild-type loci had a sequence identical to the reference sequence available in the GenBank (Accession number NC_009127.1) and all recombinant strains had the expected modified sequences. All recombinants were tested for their virulence (i.e., safety) and their ability to induce immune protection against a lethal challenge

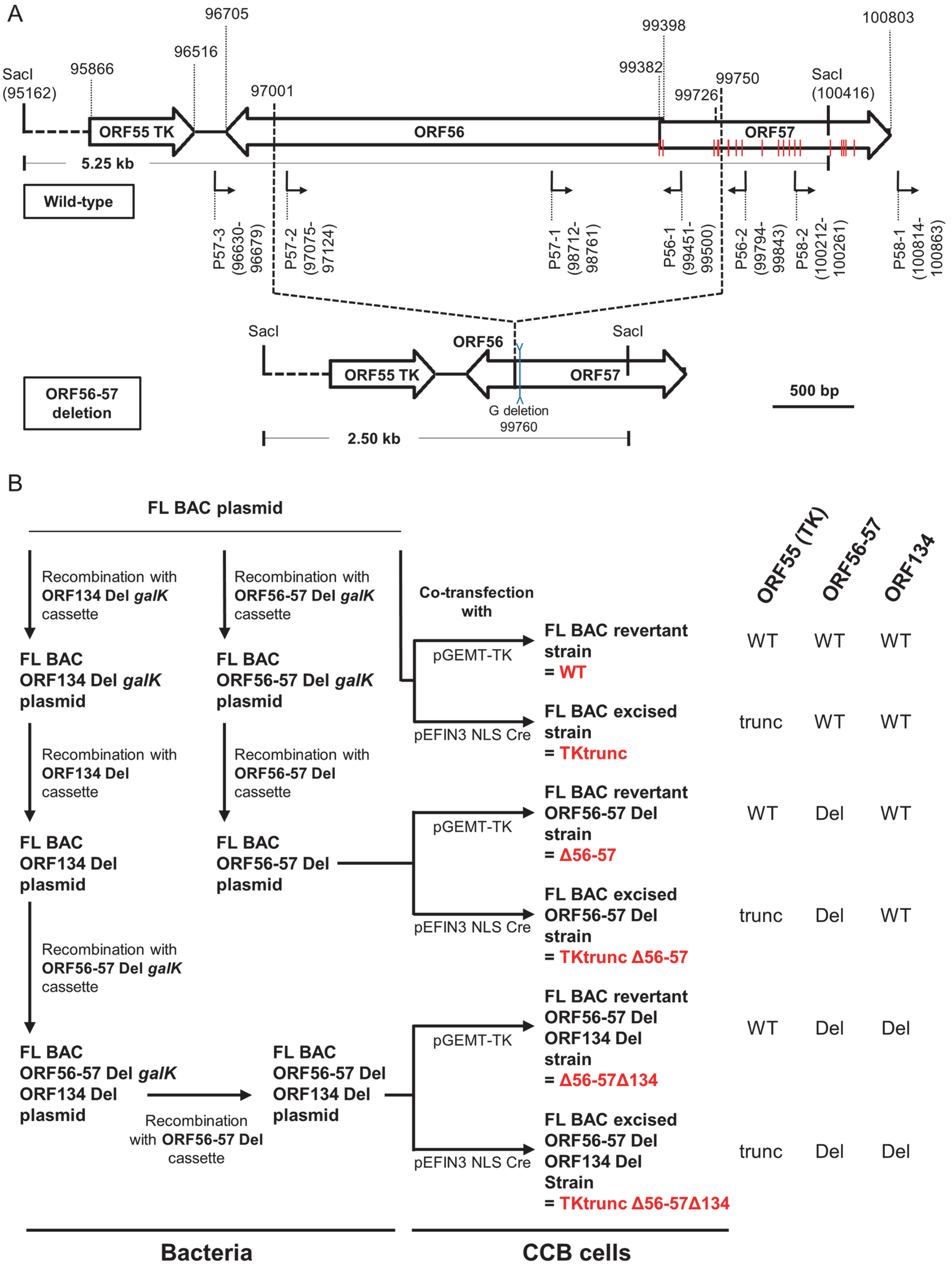

Fig 1. Production of CyHV-3 recombinants. (A) Schematic representation of wild-type ORF56-57 and ORF56-57 deleted genotypes. CyHV-3 ORFs are represented by white arrows. Predicted promoters in the ORF56-ORF57 region are represented by angular black arrows below the wild-type ORF56-57

genotype. In-frame ATGs of ORF57 are indicated by red lines. SacI restriction sites and predicted restriction fragments (in kb) are shown. Coordinates are those of the CyHV-3 reference strain available in GenBank (Accession number NC_009127.1). (B) Flowchart of the production of double (Δ56-57) and triple (Δ56-57Δ134) deleted recombinants for CyHV-3 ORF134 and/or ORF56-57. To simplify the reading of the manuscript, recombinants were given a nickname (in red) that will be used hereafter. The right side of the figure summarizes the genotype of the strains for ORF55 (TK), ORF56-57, and ORF134. Del, deleted; trunc, truncated.

doi:10.1371/journal.ppat.1004690.g001

(i.e., efficacy) (Figs. 2 and S2). Independent of the ORF55 genotype (wild-type versus truncated (TKtrunc)) and ORF134 genotype (wild-type versus deleted (Δ134)), all recombinants encoding the double ORF56-57 deletion (Δ56-57) expressed comparable safety/efficacy profiles. Fish infected with the WT (Fig. 2) or the TKtrunc (S2 Fig.) strains exhibited all clinical signs

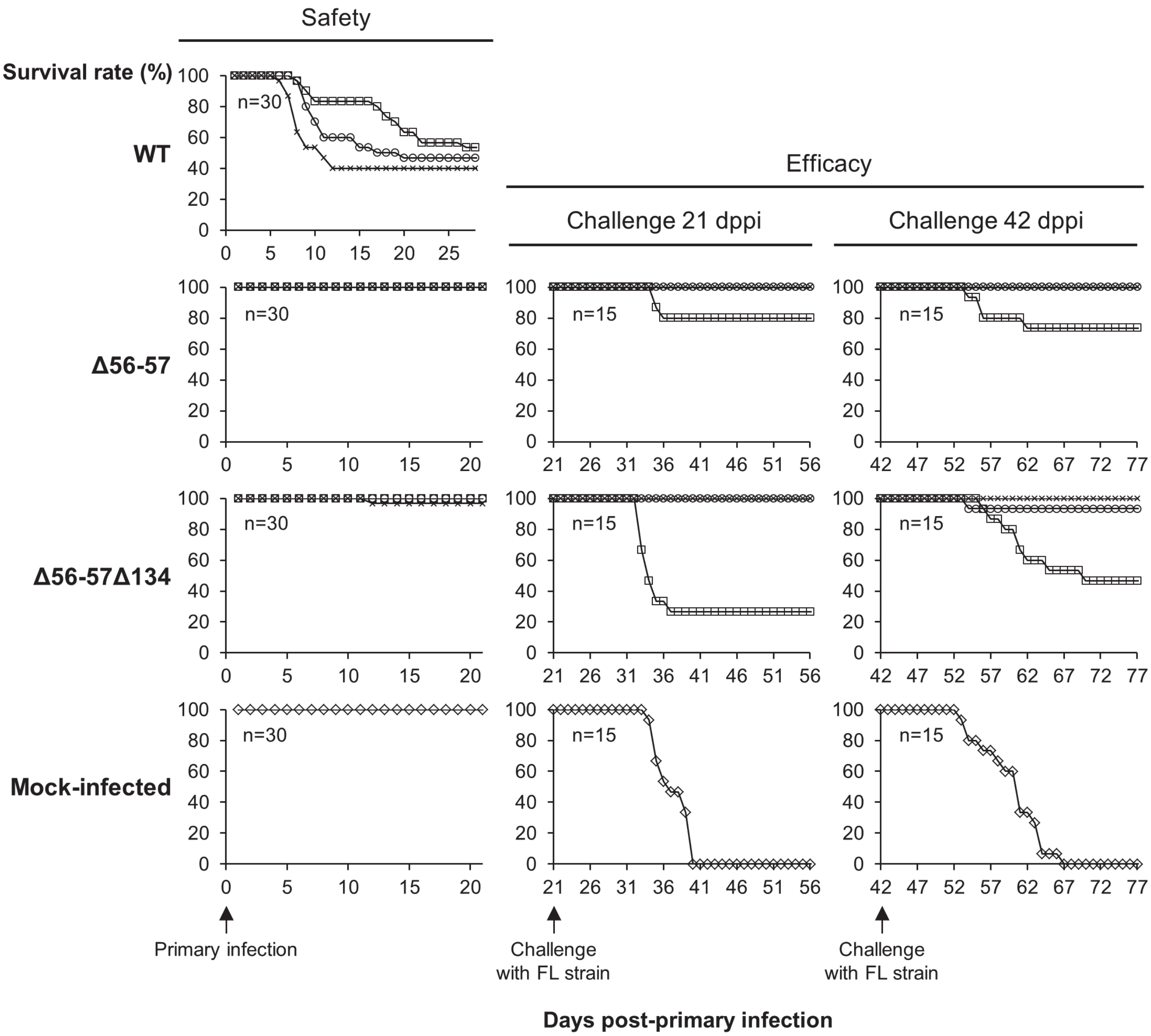

Fig 2. Safety-efficacy profile of double Δ56-57 and triple Δ56-57Δ134 deleted recombinants encoding a wild-type TK locus. The safety and efficacy of the indicated recombinant strains was tested in common carp (average weight 3.77 g ± 1.95 g, 7 months old). On day 0, fish were infected for 2 h by immersion in water containing 4 (□), 40 (○), or 400 (x) pfu/ml. Safety was investigated by measuring the survival rate for 21 days in a group of 30 carp. Efficacy was tested at 21 and 42 dppi. Mock-infected fish and fish that survived the primary infection were distributed in tanks (n = 15) and challenged by cohabitation with fish infected with the FL strain.

doi:10.1371/journal.ppat.1004690.g002

associated with CyHV-3 disease, including apathy, folding of the dorsal fin, hyperemia, increased mucus secretion, skin lesions, suffocation, erratic swimming, and loss of equilibrium. Independent of the dose tested, mortality ranged from 47 to 87%. In contrast, all double ORF56-57 deleted (Δ56-57) strains expressed an attenuated phenotype. No clinical signs were observed in fish inoculated at 4 or 40 pfu/ml, and only a few fish expressed transient mild hyperemia and folding of the dorsal fin at the higher dose (400 pfu/ml). Importantly, all fish exhibited unaltered swimming and feeding behavior. These observations were confirmed by the survival rate, which was very high in all groups (a single fish died in the group inoculated with the highest dose of the Δ56-57Δ134 strain). To investigate if the fish initially inoculated with strains encoding the ORF56-57 deletion developed a protective anti-CyHV-3 immune response, they were challenged at 21 and 42 days post-primary infection (dppi) by cohabitation with fish that were freshly inoculated with the parental FL strain (Fig. 2 and S2 Fig.). Though mock-infected fish were very sensitive to this challenge (reaching 100% mortality rate in nearly all cases), fish previously infected with strains encoding the ORF56-57 deletion at 400 pfu/ml did not express the disease and had a survival rate of 100%. Fish initially inoculated at 40 pfu/ml exhibited also very high survival rates (100% or 93%). Fish initially inoculated at 4 pfu/ml exhibited partial protection ranging from 27% to 80% survival rate. Interestingly, the challenges performed 21 dppi led to comparable results 42 dppi, indicating early onset of protective immunity. The results presented above demonstrate that the double ORF56-57 deletion correlated with a safety/efficacy profile compatible with use of the encoding strain as an attenuated vaccine. Additional truncation of ORF55 and/or deletion of ORF134 did not improve the safety/efficacy profile. The latter result is consistent with our recent study demonstrating that ORF134 does not affect CyHV-3 virulence [31] as observed for other virally encoded IL-10 homologs [32].

Taking into account the results for safety and efficacy, as well as the stability of the ORF56-57 deletion observed when the virus was passed extensively in cell culture (demonstrated by sequencing of both ORFs after passage of the virus under industrial conditions corresponding to vaccine production, Master Seed Virus +5; sequences observed were identical to initially edited sequences), the Δ56-57 strain was selected as an attenuated recombinant vaccine candidate against CyHV-3 disease.

Safety of the Δ56-57 strain studied by the IVIS

Recently, we demonstrated the usefulness of the IVIS to study the portal of entry and spreading of CyHV-3 into its host [33–36]. In the present study, we exploited this technology to investigate the safety of the Δ56-57 strain. To achieve this goal, a recombinant strain encoding both the ORF56-57 deletion and a luciferase (Luc) expression cassette inserted in a previously studied insertion site [33], hereafter called the Δ56-57 Luc strain, was produced by homologous recombination in eukaryotic cells (S3 Fig.). The molecular structure of this strain was confirmed by RFLP, Southern blot analysis (S4 Fig.), and full-length genome sequencing. Full length genome sequence of WT Luc and Δ56-57 Luc strains have been deposited in the GenBank (Accession numbers KP343683 and KP343684, respectively). The two strains share a number of defects in genes other than those noted in other strains. This includes one point deletion in ORF129 (GGGG>GGG), one inversion/deletion in ORF122, one deletion affecting the ends of ORF27 (already mutated in some other CyHV-3 strains) and ORF28. However, since these mutations are present in both WT Luc and Δ56-57 Luc strains, they are not relevant to the attenuated observed phenotype.

Before its use in vivo, the Δ56-57 Luc strain was tested in vitro. First, even if the insertion of the Luc expression cassette in a wild type strain was previously shown to have no effect on viral

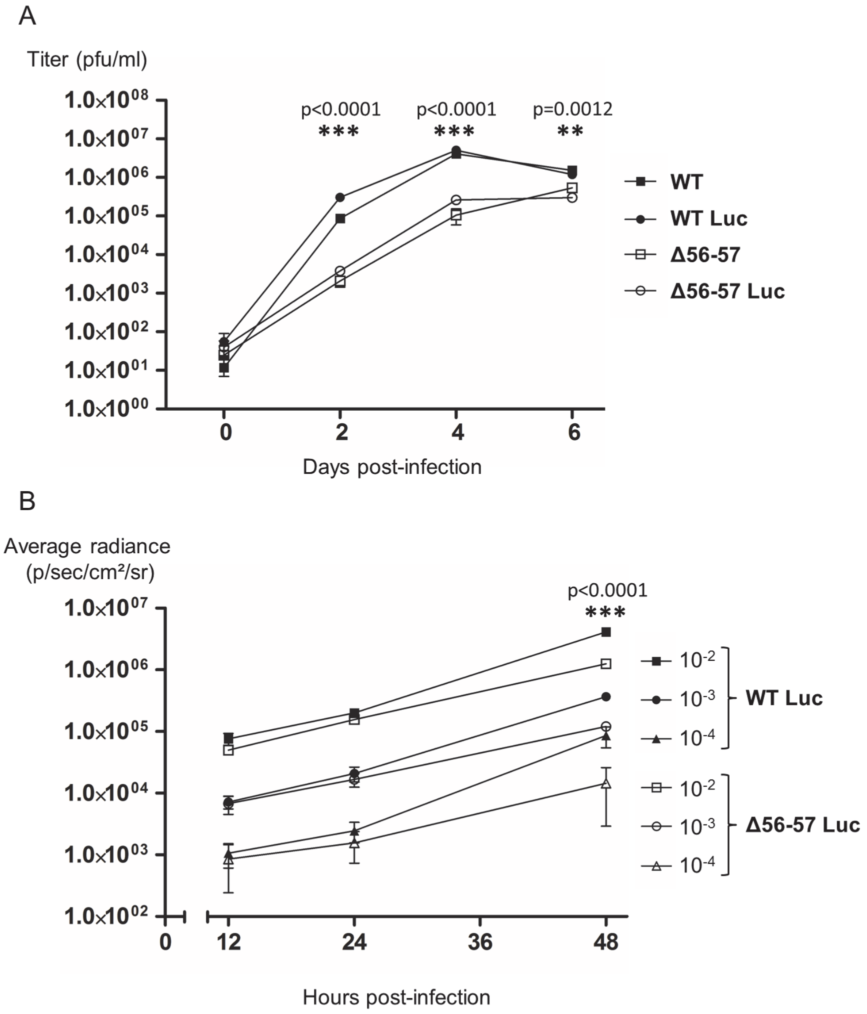

Fig 3. Effect of the double ORF56-57 deletion on CyHV-3 replication in vitro. (A) Multi-step growth curves. CCB cells were infected with the indicated strains and the viral titer (pfu/ml) in the cell supernatant determined at the indicated time points post-infection. Data presented are the mean ± SD of triplicate measurements. Significant differences between the two ORF56-57 wild-type strains (WT and WT Luc) and the two double ORF56–57 deleted strains (Δ56-57 and Δ56-57 Luc) were tested using three-way ANOVA taking ORF56–57 genotype (wild-type vs. deleted), presence or absence of Luc, and time post-infection as variables. The p-values demonstrated a significant effect of ORF56-57 genotype independent of the Luc genotype. (B) Luc activity. CCB cells were infected with the indicated strains at the indicated MOI. Luc expression was analyzed by the IVIS at different times post-infection. Data presented are the radiance ± SD of quadruplicate analyses. Significant differences were tested using three-way ANOVA taking viral strain, MOI, and time post-infection as variables. The p-values demonstrated a significant effect of the viral strain independent of the MOI. The raw data of these experiments (panels A and B) are provided online (S1 Dataset).

doi:10.1371/journal.ppat.1004690.g003

growth in cell culture and virulence in vivo [33], we tested whether this insertion affects viral growth for the Δ56-57 genotype (Fig. 3A). Replication of the Δ56-57 Luc strain was comparable to that of the Δ56-57 strain. Consistent with an earlier report [33], replication of the WT Luc strain was comparable to that of the WT strain. These data demonstrate that insertion of the Luc cassette between ORF136 and ORF137 had no effect on viral growth. However, this experiment revealed that both strains encoding the ORF56-57 deletion replicated at a significantly lower level than the strains encoding the ORF56-57 wild-type genotype ($p<0.0001$ at 2 and 4

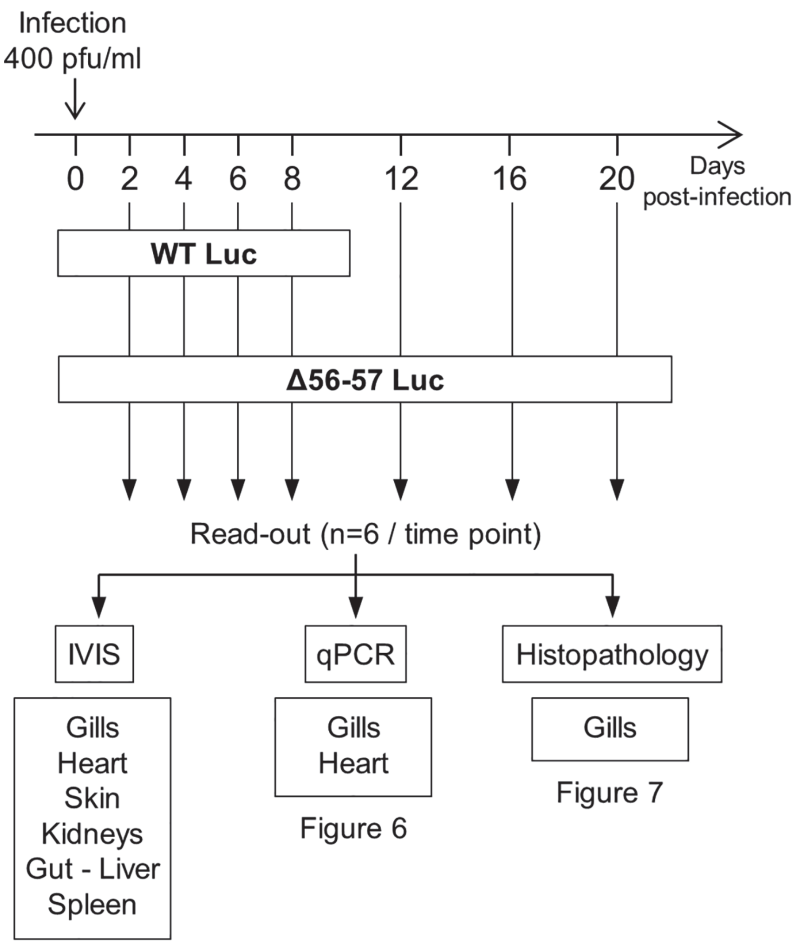

Fig 4. Flow chart of experiments performed to study the effect of the double ORF56-57 deletion on CyHV-3 pathogenesis. At time 0, carp (mean weight ± SD 30.40 g ± 9.12 g, 9 months old) were infected for 2 h by immersion in water containing WT Luc strain or the Δ56-57 Luc strain at 400 pfu/ml, then returned to larger tanks. At the indicated time points (restricted to maximum 8 dpi for the WT Luc strain to precede the peak of mortality), fish were sampled and submitted to the read-outs listed in the lower part of the figure. The read-out data are presented in Figs. 5–7. Throughout these figures, the data obtained for each fish (according to viral strain and time post-infection) are represented by the same symbol to allow correlation of the data obtained for the different organs and with the different read-outs.

doi:10.1371/journal.ppat.1004690.g004

days post-infection (dpi), and p = 0.0012 at 6 dpi). Second, to investigate whether the ORF56-57 deletion affects the expression of the Luc expression cassette, CCB cells were infected at different multiplicity of infection (MOI) with the WT Luc strain and Δ56-57 Luc strain (Fig. 3B). IVIS analyses performed 12 and 24 hours post-infection (hpi) demonstrated that the transduced Luc expression was comparable between the two strains. In contrast, analyses performed 48 hpi revealed significantly faster replication of the WT Luc strain compared to the Δ56-57 Luc strain ($p<0.0001$). Taken together, the results demonstrate that Δ56-57 Luc strain replication in vitro is comparable to that of the Δ56-57 strain, and it transduces Luc expression comparably to the WT Luc strain. These data validate the use of these two Luc recombinant strains to investigate the effect of the double ORF56-57 deletion on the pathogenesis of CyHV-3. This question was investigated by performing the experiment described in Fig. 4.

Fish were inoculated by immersion in water containing the WT Luc strain or Δ56-57 Luc strain. At different times post-infection (restricted to 8 dpi for the WT Luc strain to precede

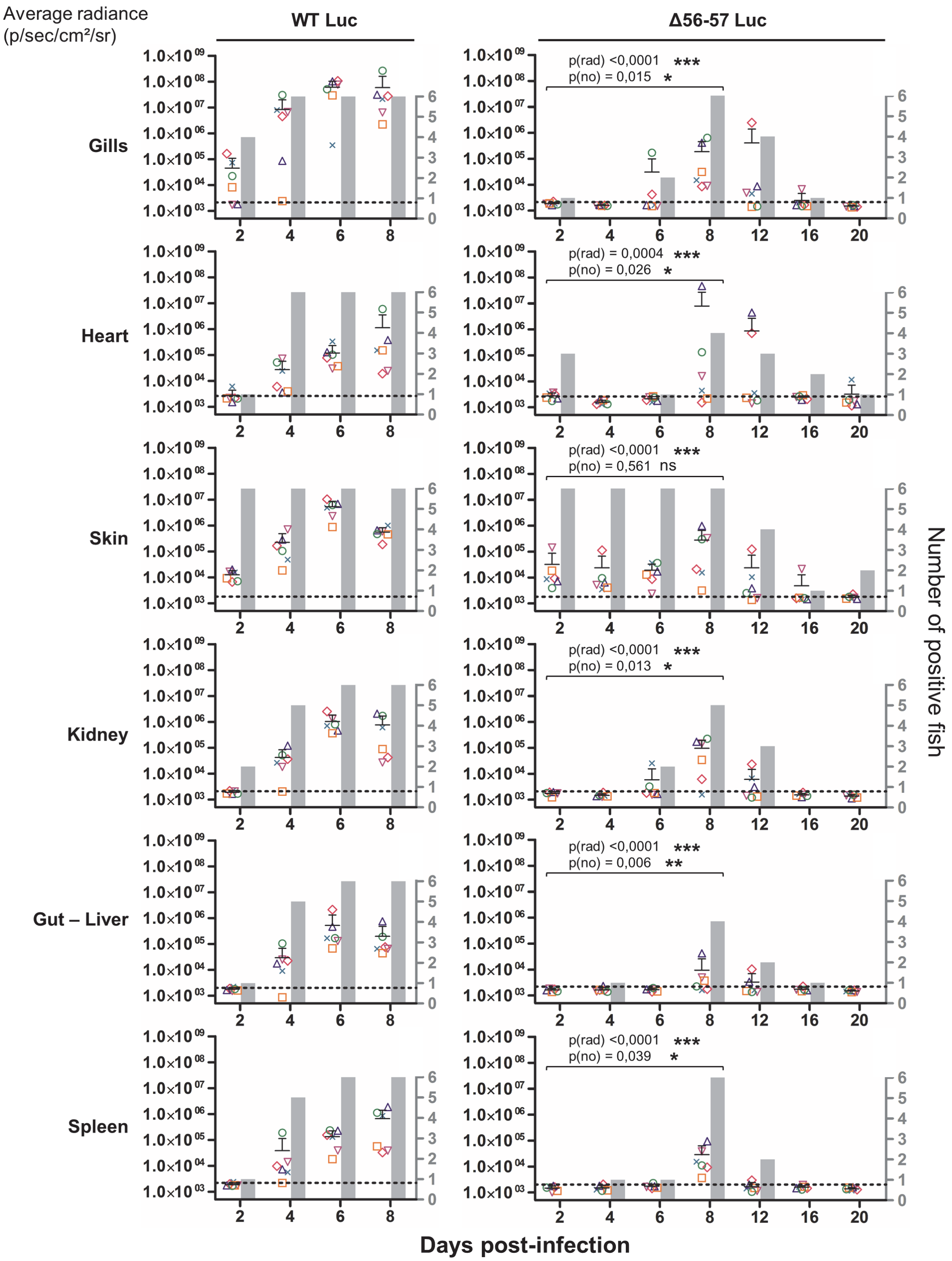

Fig 5. Effect of the double ORF56-57 deletion on viral tropism according to the IVIS. The graphs present the average radiance emitted (p/sec/cm^2/sr) per fish and per indicated organ according to time post-infection (mean + SD is represented for each time point). The average radiance was measured over a region of interest manually drawn following the outline of dissected organs. For the skin, the average radiance (individual values, mean + SD) was measured on both sides of the body, and the results for individual fish are expressed as the mean of both sides. The discontinuous line represents the cut-off for positivity and represents the mean + 3 SD ($p < 0.00135$) of the values obtained (not represented) for mock-infected fish (negative control). The number of positive fish among six analyzed fish is represented by grey bars. Statistical analyses compared the first four time points (2, 4, 6, and 8 dpi) available for both strains. The p-value and level of significance are indicated. The average emitted radiances were compared using a two-way ANOVA taking viral strain and time post-infection as variables (p(rad)), whereas the number of positive fish per group was compared using a permutation test (p(no)).

doi:10.1371/journal.ppat.1004690.g005

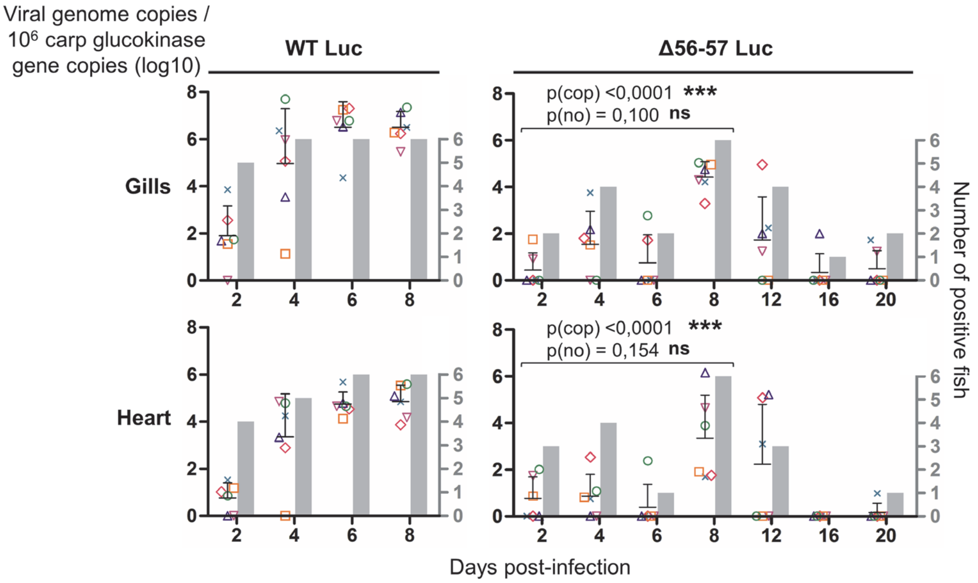

Fig 6. Effect of the double ORF56-57 deletion on viral tropism according to qPCR analysis. The number of viral genome copies is expressed as $\log_{10}$ per 10^6 carp glucokinase gene copies. Individual values represent the mean of duplicate measurements. Mock-infected fish were used as a negative control and no viral genome copies were detected in these fish. The number of positive fish among six analyzed fish is represented by grey bars. Statistical analyses compared the first four time points (2, 4, 6, and 8 dpi) available for both strains. Viral charge (Viral genome copies/10^6 carp glucokinase gene copies ($\log_{10}$)) was compared using two-way ANOVA taking viral strain and time post-infection as variables (p(cop)), whereas the number of positive fish per group was compared using a permutation test (p(no)).

doi:10.1371/journal.ppat.1004690.g006

the peak of mortality), fish were collected and each fish analyzed by the IVIS (Fig. 5), qPCR (Fig. 6), and histopathological examination (Fig. 7). The results of these read-outs were presented throughout the figures using a distinctive constant symbol for each analyzed fish according to the viral strain inoculated and the time post-infection at which the fish was collected. This mode of presentation allows identification of all results obtained for a particular fish in the figures. The skin has been shown to be the major portal of entry of CyHV-3 after inoculation by immersion in water containing the virus [33]. Consistent with this earlier report, we observed that, independent of the inoculated virus, all tested fish expressed light foci on their skin as early as 2 dpi (Fig. 5). Though the signal increased in intensity from 2 to 6 dpi on fish inoculated with the WT Luc strain, it remained stable for 12 dpi on fish inoculated with the Δ56-57 Luc strain. Global statistical analysis of the data obtained during the first 8 dpi demonstrated no significant difference in the number of positive fish between the two virus strains, as all fish were positive, but significantly less light emission was observed for fish infected with the double ORF56-57 deleted recombinant. After initial replication in the skin, the WT Luc strain spread and replicated rapidly in all tested organs, and all fish were positive in all organs at 6 dpi (Fig. 5). These results are consistent with earlier reports [33,34]. Spreading of the Δ56-57 Luc strain within infected fish was significantly delayed, reduced both quantitatively (i.e., number of positive fish) and qualitatively (i.e., intensity of the signal), and transient (Fig. 5, compare left and right columns). The reduced ability of the Δ56-57 Luc strain to spread into infected fish was further supported by qPCR analysis performed on the gills and heart (Fig. 6). Compared to fish infected with the WT Luc strain, fish infected with the double ORF56-57 deleted variant expressed significantly lower viral loads. Taken together, the results in Fig. 5 and 6 demonstrate that the Δ56-57 Luc strain was as capable of entering fish as the wild-type control strain, but it had a reduced ability to spread in the infected animal, thereby explaining its

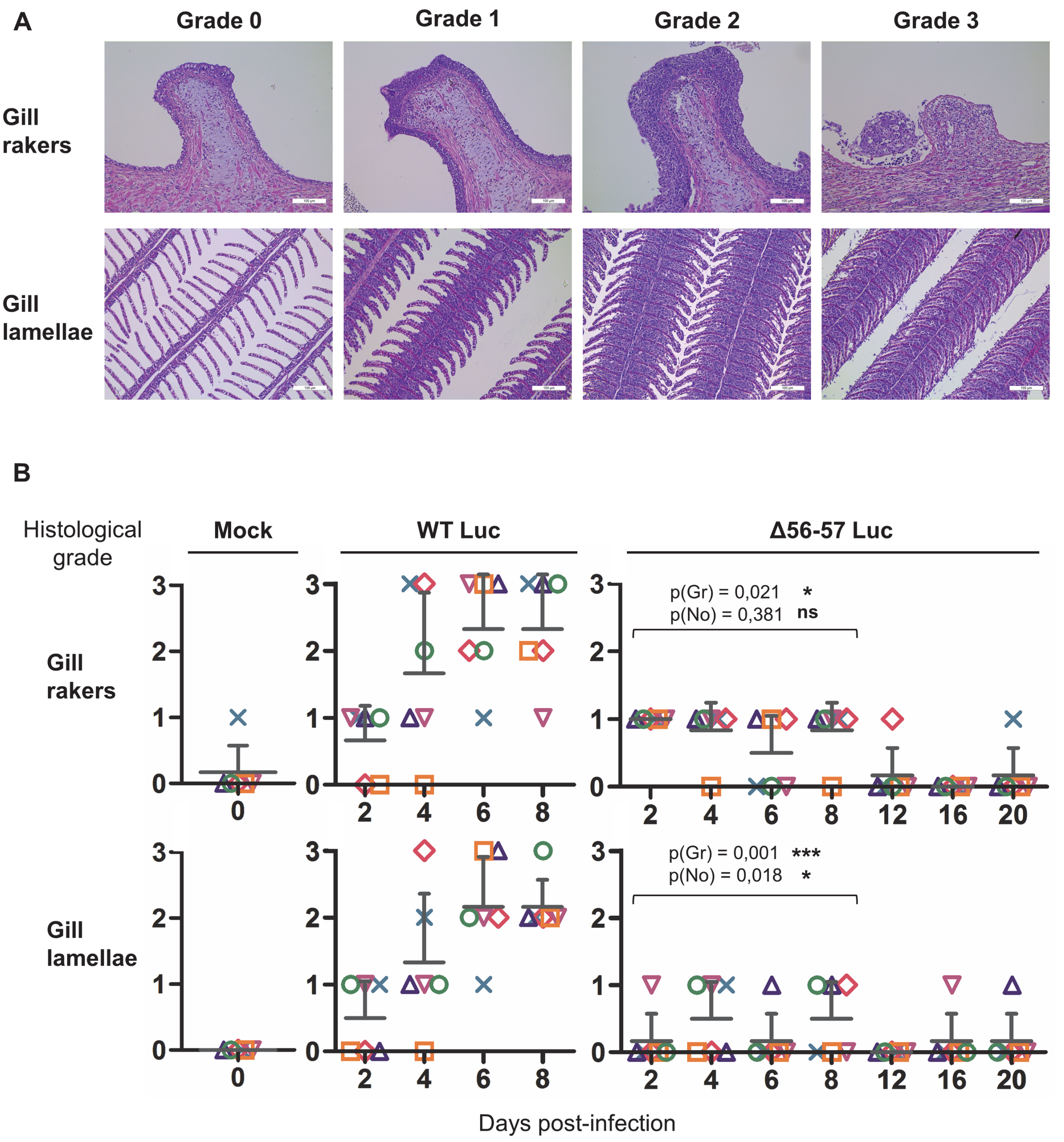

Fig 7. Effect of the double ORF56-57 deletion on viral pathogenesis based on histopathological analysis of gills. (A) Grading used to characterize the lesions observed on gill rakers and gill lamellae. Scale bars = 100 μm. (B) Histological preparations were observed by three independent observers. The grade selected by at least two of the three observers was retained as the final score. The data represent the mean grade + SD for each group at each time point. Statistical analyses compared the first four time points (2, 4, 6, and 8 dpi) available for both strains. The p-value and level of significance are indicated. Two types of statistical analyses were performed using permutation tests: comparison of the severity of the lesions (p(gr)) and comparison of the number of positive fish (p(no)) according to the viral strain.

doi:10.1371/journal.ppat.1004690.g007

attenuated phenotype. Finally, as the gills have been shown to support drastic anatomopathological modifications during CyHV-3 disease, they were examined at different times post-infection with the two viral strains (Fig. 7). Examination of both gill rakers and gill lamellae led to

the conclusion that the Δ56-57 Luc strain induced much less severe lesions than the WT Luc strain, as demonstrated by the significantly fewer positive fish (gill lamellae but not gill rakers) and significantly lower lesion scores (gill lamellae and gill rakers). These results support the attenuation conferred by the double ORF56-57 deletion and justify the selection of the Δ56-57 strain as a recombinant attenuated vaccine candidate for mass vaccination of carp by immersion in water containing the vaccine.

A key safety aspect of an attenuated recombinant vaccine is the possible spread from vaccinated to unvaccinated naïve cohabitant subjects. This aspect is particularly important for vaccination in aquaculture, where water can act as an efficient abiotic vector. To address this issue, we used the two recombinant Luc strains described above to investigate the effect of the double ORF56-57 deletion on the ability of CyHV-3 to spread from newly infected fish to naïve sentinel fish (Fig. 8). The spread of CyHV-3 was studied using two experimental settings designed to allow transmission of the virus through infectious water (water sharing) or through infectious water and physical contact between infected and naïve sentinel fish (tank sharing). Fish were infected with either the WT Luc strain or the Δ56-57 Luc strain for 2 h in water containing the virus. After rinsing in fresh water, infected fish were distributed in tanks (Fig. 8) for water sharing and tank sharing experiments. The IVIS analysis of initially infected fish 2 dpi (n = 4 for each virus strain) demonstrated that they all expressed luciferase activity on their skin as described in Fig. 5 (2 days post-infection, skin analysis), thereby demonstrating successful infection. The IVIS analysis of sentinel fish was performed after different periods of cohabitation to investigate potential spreading; the experiment was stopped at day 14 for the WT Luc strain because the vast majority of fish were dying from the infection. Under water sharing conditions, no transmission was detected for the Δ56-57 Luc strain over 18 days. In contrast, for the WT Luc strain, positive fish (2 out of 5) were detected as early as 6 days of cohabitation. Both the number and radiance of positive fish increased with time. Under tank sharing conditions, erratic and rare transmission was observed for the Δ56-57 Luc strain. Only two of the 25 analyzed fish were positive respectively after 10 and 18 days of cohabitation. In contrast, naïve sentinel fish cohabiting with fish infected with the WT Luc strain became infected as early as 6 days of cohabitation. Taken together, the results demonstrate that the double ORF56-57 deletion drastically impaired the ability of CyHV-3 to spread from freshly infected fish to naïve sentinel fish.

Efficacy of the Δ56-57 strain studied by the IVIS

The experiments presented above demonstrate the usefulness of the IVIS for studying the safety of an attenuated recombinant vaccine candidate. In the last section of this study, we also used the IVIS to characterize the immune protection conferred by the Δ56-57 strain (Fig. 9). Fish were vaccinated by immersion in water containing two different doses of the Δ56-57 strain. Six weeks post-primary infection, fish were challenged with the WT Luc strain and analyzed by the IVIS at 2, 4, and 8 days post-challenge. Analyses performed on day 2 post-challenge revealed few vaccinated fish (2 fish vaccinated at 40 pfu/ml and 1 fish vaccinated at 400 pfu/ml) expressing a low luciferase signal close to the threshold determined for the mock-infected/mock-challenged group. None of the fish analyzed on day 2 post-challenge expressed a positive signal in internal organs. No positive signal was observed on the skin or internal organs of vaccinated fish at later time points, independent of the dose used for vaccination. In contrast, mock-infected fish challenged with the WT Luc strain expressed increasing light intensity according to time post-infection. The data demonstrate that vaccination with the Δ56-57 strain induces a protective mucosal immune response at the portal of entry.

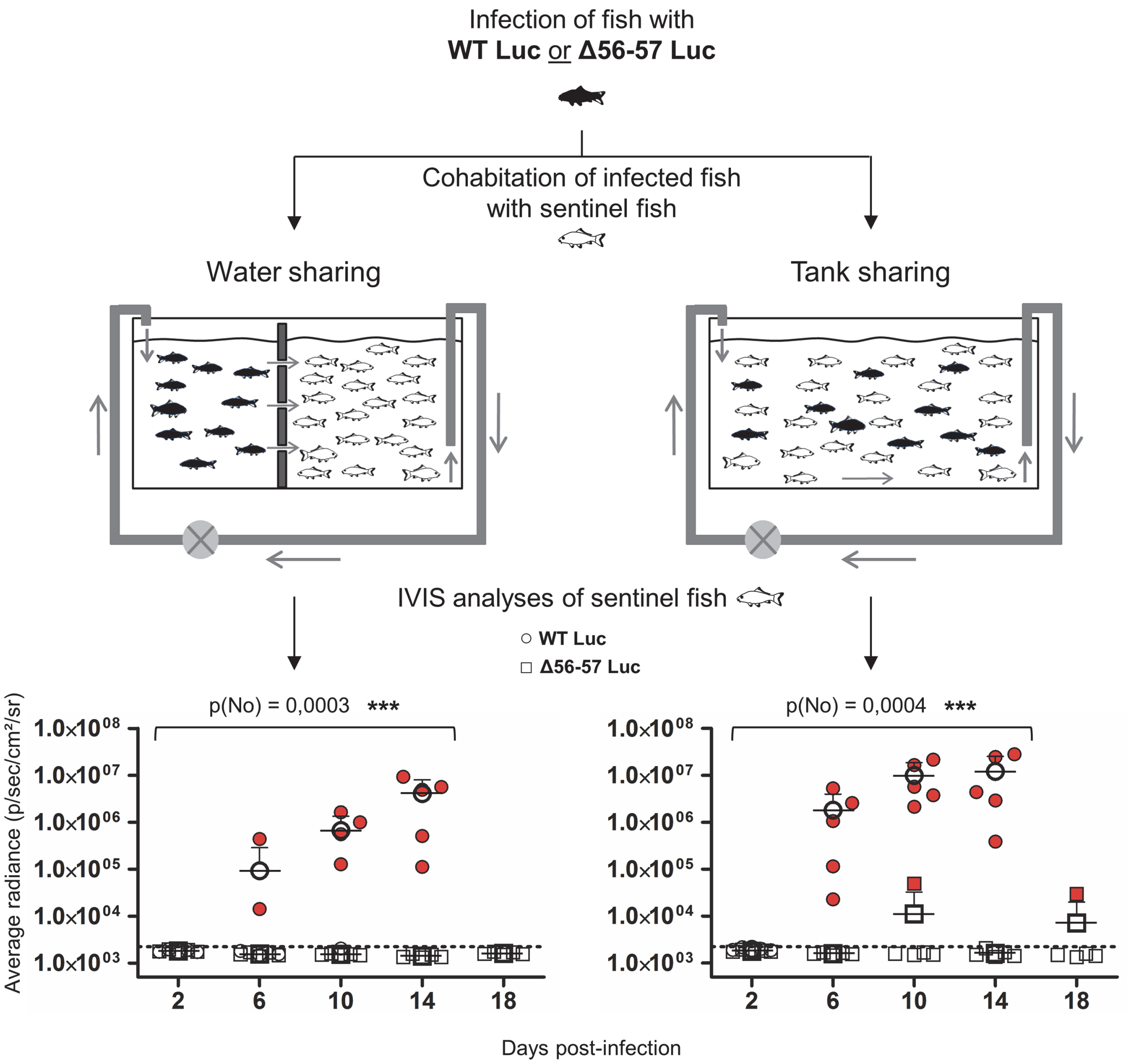

Fig 8. Effect of the double ORF56-57 deletion on CyHV-3 spread from infected fish to naïve fish. This experiment was performed using a group of common carp (mean weight ± SD 7.09 g ± 2.38 g, 9 months old). At time 0, some fish were infected (represented by black fish) by immersion for 2 h in water containing the WT Luc strain or the Δ56-57 Luc strain at 400 pfu/ml. At the end of the inoculation period, infected fish were collected, marked by cutting a fragment of the superior lobe of the caudal fin, and rinsed twice in water before distribution into two types of 60 L tanks (n = 12 per tank). The two types of tanks were designed to study virus transmission through only the water (left, water sharing) or through the water and direct fish-to-fish contact (right, tank sharing). Naive fish (represented by white fish; n = 30 per tank) were distributed to the tanks as illustrated. At the indicated time points post-infection, five naïve fish were collected from each of the four tanks (2 viral strains × 2 tank conditions) and analyzed by IVIS for Luc expression on the skin. The discontinuous line represents the cut-off for positivity ($p < 0.00135$) determined based on the analysis of mock-infected fish. Data represent the analysis of individual fish. Positive scores are presented in red. The mean radiance + SD is presented for each time point. Statistical analyses compared the number of positive fish at the first four time points (2, 4, 6, and 8 dpi) available for both strains using a permutation test. The p-value and level of significance are indicated.

doi:10.1371/journal.ppat.1004690.g008

Discussion

In the present study, we took advantage of an accidentally obtained recombinant CyHV-3 strain that exhibited a safety/efficacy profile compatible with use as an attenuated vaccine. This

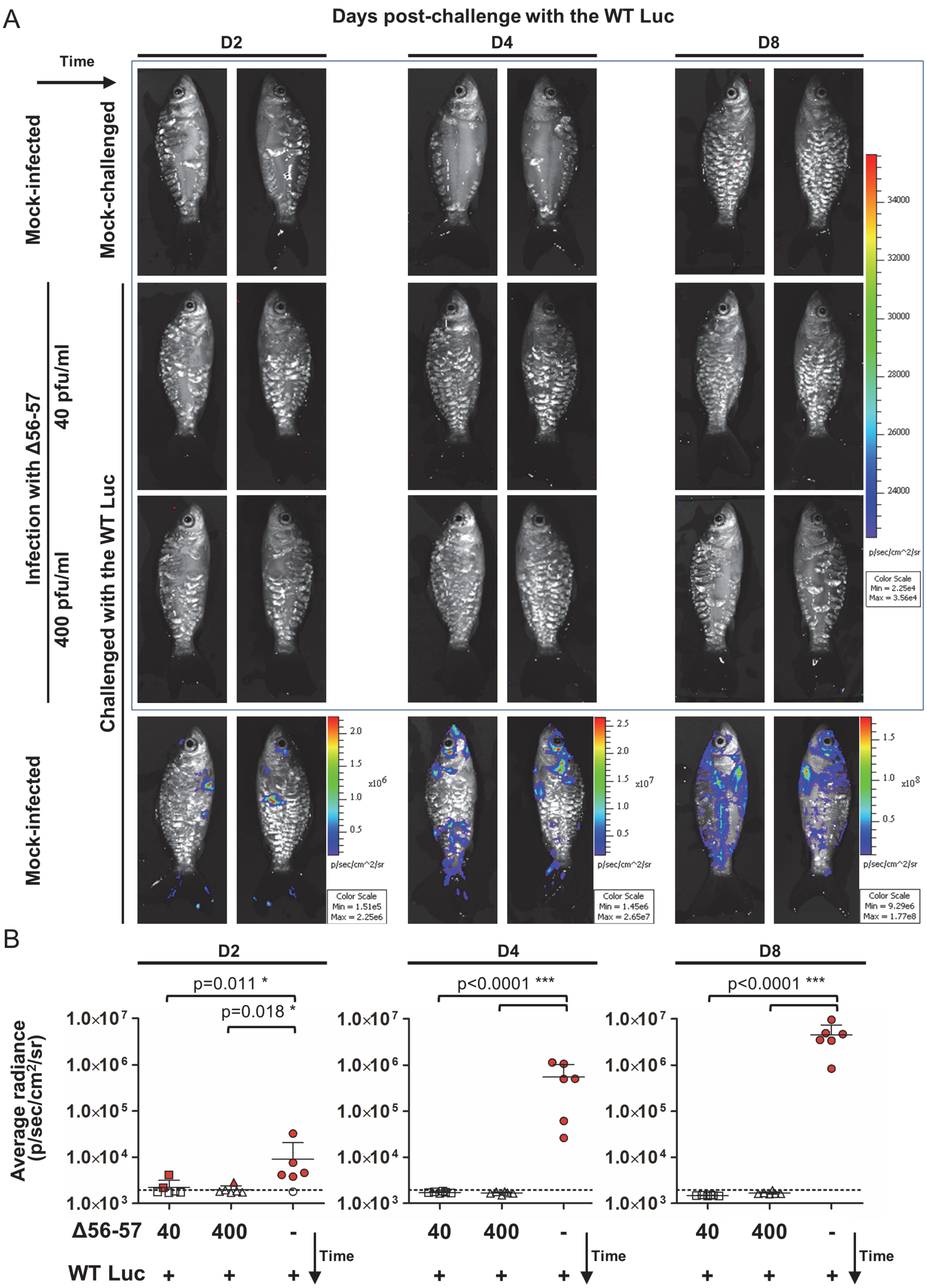

Fig 9. Immune protection conferred by the Δ56-57 strain revealed by the IVIS. Common carp (mean ± SD weight 13.82 ± 5.00 g, 9 months old) were infected for 2 h by immersion in water containing 40 or 400 pfu/ml of the Δ56–57 strain or mock-infected. None of the fish died from primary infection. Forty-two dppi, fish were challenged by immersion for 2 h in water containing 200 pfu/ml of the WT Luc strain. At the indicated time post-challenge, fish (n = 6) were

analyzed using the IVIS. (A) Representative images. Images within the blue frame were normalized using the same scale. (B) Average radiance (individual values, mean + SD) measured on the entire body surface of the fish (individual values represent the mean of the left and right sides obtained for each fish). The discontinuous line represents the cut-off for positivity, which is the mean + 3 SD ($p < 0.00135$) of the values obtained (not presented) for mock-infected and mock–challenged fish (negative control). Positive fish are represented by red filled dots. Significant differences in the mean of the average radiance were identified by post-hoc t-test after two-way ANOVA analysis taking the treatment and the time post-challenge as variables.

doi:10.1371/journal.ppat.1004690.g009

recombinant strain had deletions at three loci (ORF56-57, and ORF134). To determine the role of the double ORF56–57 deletion in the phenotype and to improve further the quality of the vaccine candidate, a series of deleted recombinants was produced and tested in vivo. The Δ56-57 strain with a deletion encompassing ORF56 and ORF57 was selected and characterized as an attenuated recombinant vaccine candidate against CyHV-3. This strain exhibited properties compatible with use as an attenuated recombinant vaccine for mass vaccination of carp by immersion in water containing the virus. It replicated efficiently in vitro, though at a lower level than the parental wild-type strain, expressed a safe attenuated phenotype, and induced a protective mucosal immune response against a lethal challenge by blocking viral infection at the portal of entry.

Deletion of ORF56 and/or ORF57 has not been reported previously for CyHV-3. Sequencing of the Cavoy attenuated anti-CyHV-3 vaccine demonstrated that it encodes a wild-type ORF56 and ORF57 sequence identical to the reference sequence available in the GenBank (Accession number NC_009127.1, sequencing from coordinates 96370 to 101248), whereas immunofluorescent staining of infected cells demonstrated the expression of both proteins (S5 Fig.). The proteins pORF56 and pORF57 have unknown functions. They both lack a signal peptide and transmembrane domain. Though pORF57 is one of the most abundant proteins of the CyHV-3 virion, pORF56 is a non-structural protein [37,38]. Extensive bioinformatic analyses did not generate a hypothesis concerning their putative functions. Homologs of CyHV-3 ORF56 and ORF57 are found in orthologous positions in the two other cyprinid herpesviruses (CyHV-1 and CyHV-2) [39], among which CyHV-2 (also known as goldfish hematopoietic necrosis virus) is responsible for a severe disease initially reported in goldfish (*Carassius auratus auratus*) that recently emerged in gibel carp (*Carassius auratus gibelio*) [40]. Based on the positional orthology of ORF56 and ORF57 in cyprinid herpesviruses, the ORF56-57 deletion reported here for CyHV-3 can likely be exploited to produce attenuated CyHV-1 and CyHV-2 recombinant vaccine candidates. Homologs of CyHV-3 ORF57 are also present in the cyprinivirus anguillid herpesvirus 1 (AngHV-1) [41] and a more distantly related virus, crocodilepox virus (CRV) [39,42], which suggests an ancestral origin for the gene. AngHV-1 infects European and Japanese eel (*Anguilla anguilla* and *Anguilla japonica*) and is responsible for mortalities of up to 30% in cultured and wild eel populations [43]. As part of a follow-up project of the present study, ORF56 and ORF57 single deleted recombinants were produced and were tested in vivo. The data obtained demonstrate that most of the attenuation observed for the double deletion ORF56-57 relied on the deletion of ORF57. These data identify the ORF57 homologue encoded by AngHV-1 (ORF35) as an obvious locus for production of an attenuated recombinant vaccine candidate.

Our earlier finding based on a wild-type CyHV-3 strain suggested that the skin is the major portal of entry after inoculation by immersion in water containing the virus [33]. After initial replication in the skin, wild-type CyHV-3 spread rapidly to virtually all organs (Fig. 5, left column). Soon after the skin, the gills, followed by other organs, support viral infection [34,44,45]. Even if the skin was always the first organ to express luciferase, the early positivity of the gills led to the hypothesis that they could represent an alternative and possibly parallel portal of entry for the virus [45–47]. The present study of the tropism of the Δ56-57 strain demonstrated that it also spreads from the skin to all tested organs. However, compared to the wild-type

strain, its systemic spread to the other organs was much slower, and its replication was reduced in intensity and duration (Figs. 5 and 6). The slower spread of the Δ56-57 vaccine strain within infected fish allowed better discrimination of the portal(s) of entry from secondary sites of infection. Though the skin of all fish was positive as early as 2 dpi, all of the other tested organs (including gills and gut) were positive in the majority of fish after 6 dpi. These data further demonstrate that the skin is the major portal of entry of CyHV-3 after infection by immersion and suggest that the other organs (including gills and gut) represent secondary sites of replication.

The double ORF56-57 deletion reduced the ability of the virus to spread within infected fish and impaired virus transmission from infected fish to naïve sentinel fish. In the present study, we designed two tank systems to test the ability of CyHV-3 strains to spread from infected to naïve sentinel subjects through indirect or indirect-and-direct contact (Fig. 8). The absence of detectable transmission under water sharing conditions and the very low level of transmission detected under tank sharing conditions suggested that fish inoculated with the vaccine candidate strain excrete very low amounts of infectious particles during the 18 days following vaccination. Combined with the observation that all vaccinated fish supported skin infection during the first 8 dpi, these data demonstrate that the ORF56-57 deletion drastically impaired viral excretion by infected subjects. Notably, the spread of the vaccine candidate strain was tested in the present study by cohabitation of fish immediately after vaccination. This is in contrast to previous studies that tested spreading by starting cohabitation weeks after vaccination, when replication of the attenuated vaccine strain was ending [27].

In addition to its relevance for applied research, the CyHV-3 - carp model has numerous qualities as a subject of fundamental research [9,11,12]. First, it is phylogenetically distant from the vast majority of herpesviruses studied so far, thereby providing an original field of research [10,39,48]. Second, it can be studied in laboratories by infection of its natural host (homologous virus-host model). Third, it allows the study of the complete infectious cycle of an alloherpesvirus, including transmission from infected to naïve animals. Transmission is an essential step in the biological cycle of pathogens and acts as a main motor of their evolution. However, very few laboratory models currently studied among members of the *Herpesvirales* allow the study of transmission [49]. Here, we report two systems allowing the study of CyHV-3 transmission by indirect or direct contact between infected and naïve sentinel fish. They will be useful for understanding the biology of an alloherpesvirus and for evaluating the ability of prophylactic strategies to inhibit the spread of wild-type strains in a fish population. The difference in transmission kinetic observed between the two systems demonstrated that direct contact between subjects promotes transmission of CyHV-3. Early replication of the virus at the portal of entry should contribute not only to the spread of the virus in infected fish, but also to the spread of the virus in the fish population. As early as 2 to 3 dpi, infected fish rubbed themselves against each other or objects. This behavior could contribute to skin-to-skin transmission [35]. Later during infection, transmission could also occur when uninfected fish peck macroscopic skin herpetic lesions developed by infected fish, thereby being infected by both skin to skin contact and infection of the pharyngeal periodontal mucosa [34]. These data highlight the importance of inducing a mucosal immune response through vaccination against CyHV-3.

Immunization of carp by immersion in water containing the vaccine candidate strain induced protective mucosal immunity, preventing replication of a challenging strain. Vaccination by immersion has several advantages. Firstly, it is compatible with mass vaccination. Secondly, in contrast to injection based vaccination, it is not restricted by a minimum fish size. Though the experiments reported in the present study were performed with fish older than 6 months, the safety and the efficacy of the Δ56-57 strain has been demonstrated in younger fish (4 months old fish, mean weight of 1.3 g). These data demonstrated that the Δ56-57 strain is

compatible with vaccination of carp soon after they acquired a competent adaptive immune system (at the end of the second month of life [50,51]). Thirdly, in contrast to the oral route, which favors the greediest fish, vaccination by immersion delivers a comparable dose to each fish. Finally, it induces antigen exposition and, hopefully, an adaptive immune response at the portal of entry used by the pathogen. The data presented in Fig. 9 demonstrate that the adaptive immune response induced by the vaccine candidate prevented infection of a challenging strain at the portal of entry. The mucosal immunity induced against CyHV-3 at the portal of entry could contribute not only to protecting vaccinated fish from CyHV-3 disease (clinical protection), but also to preventing them from transmitting virulent circulating strains (sterile immunity), thereby inducing herd immunity. This hypothesis is currently being tested using the two tank systems described above. The skin of teleost fish is a pluristratified epithelium composed exclusively of living cells covered by a mucus layer [35]. The large surface area of this mucosa, combined with its easy access, promoted the study of its innate and adaptive immune components. Interest in teleost skin as a model for studying comparative mucosal immunity recently increased with the discovery of a new immunoglobulin isotype, IgT (or IgZ) [52–54], specialized in mucosal immunity [55–58]. This specific mucosal adaptive immune response further supports the importance of antigen presentation at the pathogen's portal of entry to induce topologically adequate immune protection capable of blocking pathogen entry into the host [59,60]. Future studies are required to unravel the mechanisms underlying the mucosal immune protection conferred by the anti-CyHV-3 vaccine candidate developed in the present study. However, this study provides an original model for studying epidermal mucosal immunity against an infectious agent in teleosts.

Since it was first described in the late 1990s, CyHV-3 rapidly spread to different continents, causing severe financial losses in the common carp and koi culture industries worldwide [9]. In addition to its negative economical and societal impacts, CyHV-3 also has a negative environmental impact by affecting wild carp populations [61,62]. Thus, CyHV-3 rapidly became the subject of applied research aiming to develop diagnostic methods and a safe/efficacious vaccine. In the present study, we used BAC cloning mutagenesis and IVIS technology to develop and characterize the first rationally designed attenuated recombinant CyHV-3 vaccine compatible with mass vaccination. In addition, the present study demonstrated the importance of the CyHV-3 - carp model as an interesting and original fundamental subject of research. This model allows the study of the complete infectious cycle (including transmission from infected to naïve animals) of an alloherpesvirus by infection of its natural host. Furthermore, infection of carp by CyHV-3 represents a unique model for studying skin mucosal immunity in teleosts in response to a natural infection.

Materials and Methods

Cells and viruses

CCB cells [63] were cultured in minimum essential medium (Sigma) containing 4.5 g/L glucose (D-glucose monohydrate; Merck) and 10% fetal calf serum (FCS) as described previously [24]. The CyHV-3 FL strain was isolated from the kidney of a fish that died from CyHV-3 infection and previously used to produce the FL BAC plasmid [24]. The FL BAC revertant ORF136 Luc strain (called WT Luc in the present study) of CyHV-3 was derived from the FL BAC plasmid by prokaryotic mutagenesis [33]. The Cavoy strain was cultured from the Cavoy vaccine (Novartis).

Fish

Common carp (*Cyprinus carpio carpio*) were kept in 60 L tanks at 24°C. Water parameters were checked twice per week. Microbiological, parasitic, and clinical examinations of the fish just before the experiments demonstrated that they were healthy. All experiments were preceded by an acclimation period of at least 2 weeks.

Inoculation of fish with CyHV-3

Two modes of inoculation were used: inoculation by immersion in infectious water and inoculation by cohabitation with newly infected fish. Fish were inoculated by immersion in water (volume adapted based on fish size and fish number to use a biomass around 10%) containing the virus for 2 h under constant aeration. At the end of the incubation period, the fish were returned to 60 L tanks. For inoculation by cohabitation, newly infected fish were produced by immersion of naïve fish for 2 h in water containing 200 pfu/ml of the FL strain. At the end of the incubation period, newly infected fish were released into the tank of fish to be contaminated at a ratio of 2 newly infected fish per 15 fish to be contaminated.

Ethics statement

The experiments, maintenance and care of fish complied with the guidelines of the European Convention for the Protection of Vertebrate Animals used for Experimental and other Scientific Purposes (CETS n° 123). The animal studies were approved by the local ethics committee of the University of Liège, Belgium (Laboratory accreditation No. 1610008, protocol No. 1059). All efforts were made to minimize suffering.

Production of CyHV-3 recombinant strains using BAC cloning and prokaryotic recombination technologies

Different recombinant BAC plasmids were produced using the FL BAC [24] and FL BAC ORF134 Del plasmids [31] as parental plasmids (Fig. 1B). Recombinant plasmids were produced based on the strategy described in Fig. 1B using *galK* positive/negative selection in bacteria as described previously [64]. Recombination cassettes encoding *galK* were produced by PCR using the primers listed in Table 1 and the p*galK* vector as the template. The ORF56-57 Del cassette consisted of 250 bp upstream (coordinates 96751-97000) and 249 bp downstream (coordinates 99751-100000 with deletion of base 99760) of the ORF56-57 deletion (Fig. 1A, ORF56-57 deletion). To reconstitute the infectious virus, the recombinant BAC plasmids were co-transfected in CCB cells using polyethylenimine (3 µg polyethylenimine per 1 µg DNA) with either the pGEMT-TK plasmid or pEFIN3 NLS Cre (molecular ratio 1:75) [24]. Transfection with pGEMT-TK plasmid induced recombination upstream and downstream of the BAC cassette, leading to its complete removal and consequent reversion to a wild-type TK locus (FL BAC revertant strains). Transfection with pEFIN3 NLS Cre induced expression of a nuclear Cre recombinase and cre-loxP-mediated excision of the BAC cassette. Viruses reconstituted (FL BAC excised strains) by this procedure express a truncated form of TK due to a 172 bp foreign sequence of the BAC cassette left in the ORF55 locus. Independent of the method, EGFP-negative plaques (the BAC cassette encodes an EGFP expression cassette) were picked and amplified.

Production of the Δ56-57 Luc strain by recombination in eukaryotic cells

The Δ56-57 Luc strain was produced by co-infection of CCB cells with two parental strains (S3 Fig.). CCB cells were superinfected at the MOI of 10 pfu/cell with the WT Luc strain and Δ56-

Table 1. Primers used in this study.

	Primer name	Sequence (5'- 3')	Coordinates*/ Accession number
		**Synthesis of probes for Southern blot analysis**	
**Probe name**			
CyHV-3 ORF55	ORF55InF	AGCGCTACACCGAAGAGTCC	95990–96009
	ORF55stopR	TCACAGGATAGATATGTTACAAG	96516–96494
CyHV-3 ORF 56–57 Del	ORF56–57Pr5F	GGTACAAGACGGCCTGCTG	97247–97265
	ORF56–57Pr9R	GCCAGCACGTAGAGCTTGTG	99686–99667
CyHV-3 ORF134 Del	ORF134InF	GGTTTCTCTTTGTAGTTTTCCG	229362–229383
	ORF134InR	CACCCCAACTTTTGAGACAAC	229795–229765
CyHV-3 ORF136–137	ORF136F2	ATGAAGGCCTCTAAACTGCTG	231339–231359
	ORF137R2	ATGGACAGCACAAACGTTAC	233804–233823
Luc	LUCF3	TTGTGGATCTGGATACCGGG	
	LUCR3	GACACCTGCGTCGAAGATGT	
		**qPCR analysis**	
**Gene amplified**			
CyHV-3 ORF89	KHV-86F	GACGCCGGAGACCTTGTG	AF411803
	KHV-163R	CGGGTTCTTATTTTTGTCCTTGTT	
	KHV-109P	(6FAM) CTTCCTCTGCTCGGCGAGCACG (BHQ1)	
Carp glucokinase	CgGluc-162F	ACTGCGAGTGGAGACACATGAT	AF053332
	CgGluc-230R	TCAGGTGTGGAGCGGACAT	
	CgGluc-185P	(6FAM) AAGCCAGTGTCAAAATGCTGCCCACT (BHQ1)	
		**Synthesis of recombination cassettes**	
**Cassette name**			
ORF 134 Del *galK*	ORF134 *galK* F	ATGTTCCTTGCAGTGCTACTAACCGCGACCATCTTCTTCGAGGCTCGGGG*CCTGTTGACAATTAATCATCGGCA*	229791–229840
	ORF134 *galK* R	TCAATGTTTGCGCTTGGTTTTCATGTTCTTGACGTCTTTTGCGACCAGGA*TCAGCACTGTCCTGCTCCTT*	229217–229266
ORF 56–57 Del *galK*	ORF56–57 *galK* F	GTCCCTCGACAGCCCCAGCCCGCACAGCAGTCGCCACTCTTCCCTGTTGA*TCAGCACTGTCCTGCTCCTT*	96951–97000
	ORF56–57 *galK* R	AACCCGTACACGACGCGCTCAAGCAGCTTGATCTTGACGACGTCGTGCAC*CCTGTTGACAATTAATCATCGGCA*	99800–99751

*Coordinates based on the reference CyHV-3 genome (GenBank accession number: NC_009127.1)

Underlined: 50 bp corresponding to the CyHV-3 sequence

Italic: sequence corresponding to *galK*

doi:10.1371/journal.ppat.1004690.t001

57 strain (ratio 1:1). When all cells expressed a cytopathic effect, the supernatant containing progeny virions was collected and submitted to limiting dilution of clone virions. The recombinant viral clone expressing both Luc and ORF56-57 deletion was selected by successive screening with the IVIS and PCR genotyping, respectively.

Production of antibodies raised against pORF56 and pORF57

Mouse polyclonal antibodies (pAb) directed against the unstructured domain (IUPred, http://iupred.enzim.hu) of pORF56 encoded by coordinates 98049-99398 (GenBank accession number NC_009127.1) were produced by DNA immunization using a customized commercial service (DelphiGenetics). Mouse monoclonal antibodies (mAb) directed against pORF57 were selected from a bank of mAbs raised against CyHV-3 structural proteins; mAb 6B2 recognizes an epitope expressed in the last 165 amino acid residues of pORF57 (genomic coordinates 100309-100803, GenBank accession number NC_009127.1).

Indirect immunofluorescence staining

Cells were fixed in PBS containing 4% (w/v) paraformaldehyde at 4°C for 15 min and then 20°C for 30 min. After washing with PBS, samples were permeabilized in PBS containing 0.1% (v/v) NP-40 at 37°C for 15 min. Immunofluorescent staining (incubation and washes) was performed in PBS containing 10% FCS (v/v). Mouse pAb raised against pORF56 (diluted 1:500), mAb 6B2 raised against pORF57 (diluted 1:2500), and rabbit pAb raised against CyHV-3 structural proteins (diluted 1:1500) were used as the primary antibodies. The primary antibody was incubated at 37°C for 1 h. Alexa Fluor 488 goat anti-mouse immunoglobulin G (H+L) and Alexa Fluor 568 goat anti-rabbit immunoglobulin G (H+L) (Invitrogen) were used as the secondary antibodies. The secondary antibody was incubated at 37°C for 30 min. After washing, cells were mounted using Prolong Gold Antifade Reagent with DAPI (Invitrogen).

Genetic characterization of CyHV-3 recombinants

CyHV-3 recombinants were characterized by RFLP using SacI digestion, Southern blot analyses [31,33], sequencing of regions of interest (ROIs), and for some recombinants, full-length genome sequencing. For full-length genome sequencing, DNA (500 ng) was sheared by sonication to an average size of 400 bp and prepared for sequencing using a KAPA library preparation kit (KAPA Biosystems, Woburn, MA, USA). The fragments were A-tailed, ligated to the NEBnext Illumina adaptor (New England Biolabs, Ipswich, MA, USA), and amplified by PCR. Index tags were added by six cycles of PCR using KAPA HiFi HotStart and NEBnext indexing primers. The samples were analyzed using a MiSeq DNA sequencer running v2 chemistry (Illumina, San Diego, CA, USA). Approximately 1 million 250-nucleotide paired-end reads were obtained per sample. The reads were prepared for assembly using Trim Galore v. 0.2.2 (http://www.bioinformatics.babraham.ac.uk/projects/trim_galore). A scaffold was assembled de novo for the WT Luc strain using AbySS [65] and finished by referencing GenBank accession number NC_009127.1. Sequence accuracy was checked by assembling the reads against this sequence using BWA v. 0.6.2-r126 [66] and visualizing the alignment using Tablet v. 1.13.08.05 [67]. The other sequences were obtained by assembling the relevant reads against conceptual modifications of the sequence of the WT Luc strain using BWA and checking it using Tablet. In all viral genomes, two regions were of undetermined length: an A repeat and a GA repeat located at 32540–32465 and 177568-177730 nt, respectively, in NC_009127.1. The full length genome sequence of the WT Luc and Δ56-57 Luc strains were deposited in the GenBank (Accession numbers KP343683 and KP343684, respectively).

Multi-step growth curves

Triplicate cultures of CCB cells were infected at a MOI of 0.1 pfu/cell. After an incubation period of 2 h, cells were washed with PBS and overlaid with Dulbecco's modified essential medium (DMEM, Sigma) containing 4.5 g of glucose/L and 10% FCS. Supernatant was removed from the infected cultures at successive intervals (0, 2, 4, and 6 dpi) and stored at-80°C. The titration of infectious viral particles was determined by duplicate plaque assays in CCB cells as described previously [31,33].

In vivo bioluminescent imaging

Firefly (*Photinus pyralis*) luciferase was imaged using an IVIS (IVIS spectrum, Caliper LifeSciences) as described previously [33–35]. For cell culture analysis, the culture medium was replaced with fresh medium containing D-luciferin (150 μg/ml) (Caliper LifeSciences). Analyses were performed after an incubation period of 5 min at room temperature. For in vivo analyses, fish were anesthetized with benzocaine (25 mg/L of water). Fifteen minutes before bioluminescence analysis, D-luciferin (150 mg/kg of body weight) was injected into the peritoneal cavity. After 15 minutes, the fish were analyzed in vivo lying on their right and left sides and ex vivo after euthanasia. Dissected organs were analyzed independent from the body. All images presented in this study were acquired using a field view of C or D, a maximum auto-exposure time of 1 minute, a binning factor of 4, and a f/stop of 1. The relative intensities of transmitted light from bioluminescence and scales were determined automatically and represented as a pseudocolor image ranging from violet (least intense) to red (most intense) using Living Image 3.2 software. ROIs were drawn manually by surrounding the organs or body outline, and the average radiance (p/sec/cm^2/sr) was taken as the final measure of the bioluminescence emitted over the ROI.

Quantification of virus genome copies in organs by real-time TaqMan PCR

The virus genome was quantified by real-time TaqMan PCR as described previously [31], by amplifying fragments of the CyHV-3 ORF89 and carp glucokinase genes. The primers and probes are listed in Table 1.

Histopathological analysis of gills

Gills were dissected immediately after euthanasia and fixed in 4% buffered formalin before embedding in paraffin [31]. Five-micrometer sections were stained with hematoxylin and eosin, mounted, and examined by microscopy. Three independent observers scored the lesions in a blind test mode using a 4-step scale. For each sample, a score was attributed for the gill rakers and gill lamellae. When at least two observers agreed on a grade, the corresponding grade was attributed. In a few exceptional cases, when all three examiners scored differently, additional analyses were performed until achieving a majority. The grading system evaluated the degree of epithelial hyperplasia, the presence of intra-nuclear viral inclusions, and cell degeneration (Fig. 7A). Briefly, grade 0 = physiological state; grade 1 = mild hyperplasia without evidence of degenerated cells and viral inclusions; grade 2 = severe hyperplasia and presence of few degenerated cells and viral inclusions; and grade 3 = the presence of abundant degenerated cells and viral inclusions (gill lamellae and gill rakers), massive epithelial hyperplasia filling the entire secondary lamellae inter-space (gill lamellae), and ulcerative erosion of the epithelium (gill rakers).

Statistical analysis

The number of positive fish or positive organs (qualitative data) according to the two viral strains was compared by a permutation test (Figs. 5–8). Briefly, all occurrences were recorded in a dataset (observed dataset). A series of 10,000 random repetitions of the same procedure was performed to allocate positive events to the two groups, creating the shuffled datasets. For each dataset (observed dataset and shuffled datasets), the global difference between the two groups was calculated by summing the daily observed differences. The proportion of shuffled datasets with a global difference greater or equal to the global difference in the observed dataset was then taken as the p-value. Histopathological grading results were compared by a permutation test after calculating the value per group and per day corresponding to the sum of all individual grades obtained in each group (Fig. 7). Viral growth (Fig. 3A), IVIS (Figs. 3B, 5, and 9B), and qPCR results (Fig. 6) were compared as quantitative data (level of positivity) using two- or three-way ANOVA with interactions followed by post-hoc t-test. The variables used and comparisons retained for statistical illustrations are described in their respective figure legends. Presence or absence of statistical significance is represented as follows, ns: not significant, $* \ p<0.05$, $** \ p<0.01$, $*** \ p<0.001$.

Supporting Information

S1 Fig. Structural analysis of double Δ56-57 and triple Δ56-57Δ134 deleted recombinants. The indicated strains were analyzed by SacI restriction (left) and Southern blotting using ORF55, ORF56-57 Del, and ORF134 Del probes. Black and white arrowheads indicate fragments containing ORF134 and ORF56-57, respectively. Markers sizes (MS) are indicated on the left.
(TIF)

S2 Fig. Safety-efficacy profile of double Δ56-57 and triple Δ56-57Δ134 deleted recombinants encoding a truncated TK locus. The safety and efficacy of the indicated recombinant strains was tested in common carp (average weight 4.41 g ± 1.78 g, 7 months old). On day 0, fish were infected for 2 h by immersion in water containing 4 (□), 40 (○), or 400 (x) pfu/ml. Safety was investigated by measuring the survival rate for 21 days in a group of 30 carp. Efficacy was tested at 21 and 42 dppi. Mock-infected fish and fish that survived the primary infection were distributed in tanks (n = 15) and challenged by cohabitation with fish infected with the FL strain.
(TIF)

S3 Fig. Production of a recombinant Δ56-57 strain expressing Luciferase. (A) Molecular structure of the Δ56-57 strain and WT Luc strain used as parental strains for production of the Δ56-57 Luc strain by co-infection in eukaryotic cells. ORFs are represented by white or grey arrows, the Luc cassette by a grey rectangle, and CyHV-3 terminal repeats by white rectangles. The positions of SacI restriction sites and restriction fragment lengths are shown below each genotype. (B) Schematic representation of the method used to produce recombinant strains by co-infection of two parental strains. The molecular structures of the four possible recombinant strains resulting from unique or multiple cross-overs are illustrated.
(TIF)

S4 Fig. Structure analysis of the Δ56-57 Luc strain. The indicated strains were analyzed by SacI restriction (left) and Southern blotting using ORF55, ORF56-57 Del, ORF136-137, and Luc probes. White and white-outlined black arrowheads indicate fragments containing ORF56-57 loci and ORF136-137 loci, respectively. Black arrowheads indicate the restriction

fragment containing most of the Luc cassette sequence. Marker sizes (MS) are indicated on the left.
(TIF)

S5 Fig. pORF56 and pORF57 expression by the Cavoy strain of CyHV-3. CCB cells were infected with the indicated strains. One day post-infection, cells were treated for indirect immunofluorescent staining and confocal microscopic analysis of pORF56 or pORF57 (green), CyHV-3 structural proteins (red), and cell nuclei (blue). The overlay represents the superposition of the three channels. White scale bars = 20 μm.
(TIF)

S1 Dataset. Raw data of the experiments presented in Fig. 3.
(XLSX)

Acknowledgments

The authors are grateful to Cédric Delforge, Emeline Deglaire, Justine Javaux, and Lorène Dams for their excellent technical assistance.

Author Contributions

Conceived and designed the experiments: MB MR AV. Performed the experiments: MB MR PO GF KR GSW FL HL AV. Analyzed the data: MB MR FF CB DD AJD AV. Contributed reagents/materials/analysis tools: AR KR GSW FL HL. Wrote the paper: MB MR FF CB AJD AV.

References

1. F.A.O. (2014) The State of World Fisheries and Aquaculture 2014. Rome. 223 p.
2. Muir J (2005) Managing to harvest? Perspectives on the potential of aquaculture. Philos Trans R Soc Lond B Biol Sci 360: 191–218. PMID: 15713597
3. Bostock J, McAndrew B, Richards R, Jauncey K, Telfer T, et al. (2010) Aquaculture: global status and trends. Philos Trans R Soc Lond B Biol Sci 365: 2897–2912. doi: 10.1098/rstb.2010.0170 PMID: 20713392
4. Salgado-Miranda C, Loza-Rubio E, Rojas-Anaya E, Garcia-Espinosa G (2013) Viral vaccines for bony fish: past, present and future. Expert Rev Vaccines 12: 567–578. doi: 10.1586/erv.13.38 PMID: 23659303
5. Sommerset I, Krossoy B, Biering E, Frost P (2005) Vaccines for fish in aquaculture. Expert Rev Vaccines 4: 89–101. PMID: 15757476
6. Rodgers CJ, Mohan CV, Peeler EJ (2011) The spread of pathogens through trade in aquatic animals and their products. Rev Sci Tech 30: 241–256. PMID: 21809767
7. Balon EK (1995) Origin and domestication of the wild carp, *Cyprinus carpio*: from Roman gourmets to the swimming flowers. Aquaculture 129: 3–48.
8. F.A.O. Global Aquaculture Production 1950–2012. Available http://www.fao.org/fishery/statistics/global-aquaculture-production/query/fr/en/. Accessed 22 May 2014.
9. Rakus K, Ouyang P, Boutier M, Ronsmans M, Reschner A, et al. (2013) Cyprinid herpesvirus 3: an interesting virus for applied and fundamental research. Vet Res 44: 85. doi: 10.1186/1297-9716-44-85 PMID: 24073814
10. Waltzek TB, Kelley GO, Stone DM, Way K, Hanson L, et al. (2005) Koi herpesvirus represents a third cyprinid herpesvirus (CyHV-3) in the family Herpesviridae. J Gen Virol 86: 1659–1667. PMID: 15914843
11. Michel B, Fournier G, Lieffrig F, Costes B, Vanderplasschen A (2010) Cyprinid herpesvirus 3. Emerg Infect Dis 16: 1835–1843. doi: 10.3201/eid1612.100593 PMID: 21122210
12. Adamek M, Steinhagen D, Irnazarow I, Hikima J, Jung TS, et al. (2014) Biology and host response to Cyprinid herpesvirus 3 infection in common carp. Dev Comp Immunol 43: 151–159. doi: 10.1016/j.dci.2013.08.015 PMID: 23981329

13. Bondad-Reantaso MG, Subasinghe RP, Arthur JR, Ogawa K, Chinabut S, et al. (2005) Disease and health management in Asian aquaculture. Vet Parasitol 132: 249–272. PMID: 16099592

14. Sunarto A, Rukyani A, Itami T (2005) Indonesian Experience on the Outbreak of Koi Herpesvirus in Koi and Carp (*Cyprinus carpio*). Bull Fish Res Agen Supplement 2: 15–21.

15. Brudeseth BE, Wiulsrod R, Fredriksen BN, Lindmo K, Lokling KE, et al. (2013) Status and future perspectives of vaccines for industrialised fin-fish farming. Fish Shellfish Immunol 35: 1759–1768. doi: 10.1016/j.fsi.2013.05.029 PMID: 23769873

16. Plant KP, Lapatra SE (2011) Advances in fish vaccine delivery. Dev Comp Immunol 35: 1256–1262. doi: 10.1016/j.dci.2011.03.007 PMID: 21414351

17. Lorenzen N, LaPatra SE (2005) DNA vaccines for aquacultured fish. Rev Sci Tech 24: 201–213. PMID: 16110889

18. Perelberg A, Ilouze M, Kotler M, Steinitz M (2008) Antibody response and resistance of *Cyprinus carpio* immunized with cyprinid herpes virus 3 (CyHV-3). Vaccine 26: 3750–3756. doi: 10.1016/j.vaccine.2008.04.057 PMID: 18534721

19. Perelberg A, Ronen A, Hutoran M, Smith Y, Kotler M (2005) Protection of cultured *Cyprinus carpio* against a lethal viral disease by an attenuated virus vaccine. Vaccine 23: 3396–3403. PMID: 15837363

20. Ronen A, Perelberg A, Abramowitz J, Hutoran M, Tinman S, et al. (2003) Efficient vaccine against the virus causing a lethal disease in cultured *Cyprinus carpio*. Vaccine 21: 4677–4684. PMID: 14585675

21. Zhou J, Xue J, Wang Q, Zhu X, Li X, et al. (2014) Vaccination of plasmid DNA encoding ORF81 gene of CJ strains of KHV provides protection to immunized carp. In Vitro Cell Dev Biol Anim 50: 489–495. doi: 10.1007/s11626-014-9737-2 PMID: 24619212

22. Zhou JX, Wang H, Li XW, Zhu X, Lu WL, et al. (2014) Construction of KHV-CJ ORF25 DNA vaccine and immune challenge test. J Fish Dis 37: 319–325. doi: 10.1111/jfd.12105 PMID: 23962282

23. Fuchs W, Fichtner D, Bergmann SM, Mettenleiter TC (2011) Generation and characterization of koi herpesvirus recombinants lacking viral enzymes of nucleotide metabolism. Arch Virol 156: 1059–1063. doi: 10.1007/s00705-011-0953-8 PMID: 21387205

24. Costes B, Fournier G, Michel B, Delforge C, Raj VS, et al. (2008) Cloning of the koi herpesvirus genome as an infectious bacterial artificial chromosome demonstrates that disruption of the thymidine kinase locus induces partial attenuation in *Cyprinus carpio koi*. J Virol 82: 4955–4964. doi: 10.1128/JVI.00211-08 PMID: 18337580

25. Yasumoto S, Kuzuya Y, Yasuda M, Yoshimura T, Miyazaki T (2006) Oral Immunization of Common Carp with a Liposome Vaccine Fusing Koi Herpesvirus Antigen. Fish Pathol 41: 141–145.

26. O'Connor MR, Farver TB, Malm KV, Yun SC, Marty GD, et al. (2014) Protective immunity of a modified-live cyprinid herpesvirus 3 vaccine in koi (*Cyprinus carpio koi*) 13 months after vaccination. Am J Vet Res 75: 905–911. doi: 10.2460/ajvr.75.10.905 PMID: 25255180

27. Weber EP 3rd, Malm KV, Yun SC, Campbell LA, Kass PH, et al. (2014) Efficacy and safety of a modified-live cyprinid herpesvirus 3 vaccine in koi (*Cyprinus carpio koi*) for prevention of koi herpesvirus disease. Am J Vet Res 75: 899–904. doi: 10.2460/ajvr.75.10.899 PMID: 25255179

28. Meeusen EN, Walker J, Peters A, Pastoret PP, Jungersen G (2007) Current status of veterinary vaccines. Clin Microbiol Rev 20: 489–510. PMID: 17630337

29. Lauring AS, Jones JO, Andino R (2010) Rationalizing the development of live attenuated virus vaccines. Nat Biotechnol 28: 573–579. doi: 10.1038/nbt.1635 PMID: 20531338

30. Rueckert C, Guzman CA (2012) Vaccines: from empirical development to rational design. PLoS Pathog 8: e1003001. doi: 10.1371/journal.ppat.1003001 PMID: 23144616

31. Ouyang P, Rakus K, Boutier M, Reschner A, Leroy B, et al. (2013) The IL-10 homologue encoded by cyprinid herpesvirus 3 is essential neither for viral replication in vitro nor for virulence in vivo. Vet Res 44: 53. doi: 10.1186/1297-9716-44-53 PMID: 23865540

32. Ouyang P, Rakus K, van Beurden SJ, Westphal AH, Davison AJ, et al. (2014) IL-10 encoded by viruses: a remarkable example of independent acquisition of a cellular gene by viruses and its subsequent evolution in the viral genome. J Gen Virol 95: 245–262. doi: 10.1099/vir.0.058966-0 PMID: 24225498

33. Costes B, Raj VS, Michel B, Fournier G, Thirion M, et al. (2009) The major portal of entry of koi herpesvirus in *Cyprinus carpio* is the skin. J Virol 83: 2819–2830. doi: 10.1128/JVI.02305-08 PMID: 19153228

34. Fournier G, Boutier M, Stalin Raj V, Mast J, Parmentier E, et al. (2012) Feeding *Cyprinus carpio* with infectious materials mediates cyprinid herpesvirus 3 entry through infection of pharyngeal periodontal mucosa. Vet Res 43: 6. doi: 10.1186/1297-9716-43-6 PMID: 22276598

35. Raj VS, Fournier G, Rakus K, Ronsmans M, Ouyang P, et al. (2011) Skin mucus of *Cyprinus carpio* inhibits cyprinid herpesvirus 3 binding to epidermal cells. Vet Res 42: 92. doi: 10.1186/1297-9716-42-92 PMID: 21816061

36. Ronsmans M, Boutier M, Rakus K, Farnir F, Desmecht D, et al. (2014) Sensitivity and permissivity of *Cyprinus carpio* to cyprinid herpesvirus 3 during the early stages of its development: importance of the epidermal mucus as an innate immune barrier. Vet Res 45: 100. PMID: 25281322

37. Michel B, Leroy B, Stalin Raj V, Lieffrig F, Mast J, et al. (2010) The genome of cyprinid herpesvirus 3 encodes 40 proteins incorporated in mature virions. J Gen Virol 91: 452–462. doi: 10.1099/vir.0.015198-0 PMID: 19846671

38. Yi Y, Zhang H, Lee X, Weng S, He J, et al. (2014) Extracellular virion proteins of two Chinese CyHV-3/KHV isolates, and identification of two novel envelope proteins. Virus Res doi: 10.1016/j.virusres.2014.07.034

39. Davison AJ, Kurobe T, Gatherer D, Cunningham C, Korf I, et al. (2013) Comparative genomics of carp herpesviruses. J Virol 87: 2908–2922. doi: 10.1128/JVI.03206-12 PMID: 23269803

40. Xu J, Zeng L, Zhang H, Zhou Y, Ma J, et al. (2013) Cyprinid herpesvirus 2 infection emerged in cultured gibel carp, *Carassius auratus gibelio* in China. Vet Microbiol 166: 138–144. doi: 10.1016/j.vetmic.2013.05.025 PMID: 23850442

41. van Beurden SJ, Bossers A, Voorbergen-Laarman MH, Haenen OL, Peters S, et al. (2010) Complete genome sequence and taxonomic position of anguillid herpesvirus 1. J Gen Virol 91: 880–887. doi: 10.1099/vir.0.016261-0 PMID: 20016040

42. Afonso CL, Tulman ER, Delhon G, Lu Z, Viljoen GJ, et al. (2006) Genome of crocodilepox virus. J Virol 80: 4978–4991. PMID: 16641289

43. van Beurden SJ, Engelsma MY, Roozenburg I, Voorbergen-Laarman MA, van Tulden PW, et al. (2012) Viral diseases of wild and farmed European eel *Anguilla anguilla* with particular reference to the Netherlands. Dis Aquat Organ 101: 69–86. doi: 10.3354/dao02501 PMID: 23047193

44. Gilad O, Yun S, Zagmutt-Vergara FJ, Leutenegger CM, Bercovier H, et al. (2004) Concentrations of a Koi herpesvirus (KHV) in tissues of experimentally infected *Cyprinus carpio koi* as assessed by real-time TaqMan PCR. Dis Aquat Organ 60: 179–187. PMID: 15521316

45. Pikarsky E, Ronen A, Abramowitz J, Levavi-Sivan B, Hutoran M, et al. (2004) Pathogenesis of acute viral disease induced in fish by carp interstitial nephritis and gill necrosis virus. J Virol 78: 9544–9551. PMID: 15308746

46. Miyazaki T, Kuzuya Y, Yasumoto S, Yasuda M, Kobayashi T (2008) Histopathological and ultrastructural features of Koi herpesvirus (KHV)-infected carp *Cyprinus carpio*, and the morphology and morphogenesis of KHV. Dis Aquat Organ 80: 1–11. doi: 10.3354/dao01929 PMID: 18714678

47. Monaghan SJ, Thompson KD, Adams A, Kempter J, Bergmann SM (2014) Examination of the early infection stages of koi herpesvirus (KHV) in experimentally infected carp, *Cyprinus carpio* L. using in situ hybridization. J Fish Dis doi: 10.1111/jfd.12260

48. Waltzek TB, Kelley GO, Alfaro ME, Kurobe T, Davison AJ, et al. (2009) Phylogenetic relationships in the family Alloherpesviridae. Dis Aquat Organ 84: 179–194. doi: 10.3354/dao02023 PMID: 19565695

49. Francois S, Vidick S, Sarlet M, Desmecht D, Drion P, et al. (2013) Illumination of murine gammaherpesvirus-68 cycle reveals a sexual transmission route from females to males in laboratory mice. PLoS Pathog 9: e1003292. doi: 10.1371/journal.ppat.1003292 PMID: 23593002

50. Zapata A, Diez B, Cejalvo T, Gutiérrez-de Frías C, Cortés A (2006) Ontogeny of the immune system of fish. Fish Shellfish Immunol 20: 126–136. PMID: 15939627

51. Huttenhuis HBT, Grou CPO, Taverne-Thiele AJ, Taverne N, Rombout JHWM (2006) Carp (*Cyprinus carpio* L.) innate immune factors are present before hatching. Fish Shellfish Immunol 20: 586–596. PMID: 16169251

52. Danilova N, Bussmann J, Jekosch K, Steiner LA (2005) The immunoglobulin heavy-chain locus in zebrafish: identification and expression of a previously unknown isotype, immunoglobulin Z. Nat Immunol 6: 295–302. PMID: 15685175

53. Hansen JD, Landis ED, Phillips RB (2005) Discovery of a unique Ig heavy-chain isotype (IgT) in rainbow trout: Implications for a distinctive B cell developmental pathway in teleost fish. Proc Natl Acad Sci U S A 102: 6919–6924. PMID: 15863615

54. Ryo S, Wijdeven RH, Tyagi A, Hermsen T, Kono T, et al. (2010) Common carp have two subclasses of bonyfish specific antibody IgZ showing differential expression in response to infection. Dev Comp Immunol 34: 1183–1190. doi: 10.1016/j.dci.2010.06.012 PMID: 20600275

55. Xu Z, Parra D, Gomez D, Salinas I, Zhang YA, et al. (2013) Teleost skin, an ancient mucosal surface that elicits gut-like immune responses. Proc Natl Acad Sci U S A 110: 13097–13102. doi: 10.1073/pnas.1304319110 PMID: 23884653

56. Zhang YA, Salinas I, Li J, Parra D, Bjork S, et al. (2010) IgT, a primitive immunoglobulin class specialized in mucosal immunity. Nat Immunol 11: 827–835. doi: 10.1038/ni.1913 PMID: 20676094
57. Sunyer JO (2013) Fishing for mammalian paradigms in the teleost immune system. Nat Immunol 14: 320–326. doi: 10.1038/ni.2549 PMID: 23507645
58. Tacchi L, Musharrafieh R, Larragoite ET, Crossey K, Erhardt EB, et al. (2014) Nasal immunity is an ancient arm of the mucosal immune system of vertebrates. Nature communications 5: 5205. doi: 10.1038/ncomms6205 PMID: 25335508
59. Gomez D, Sunyer JO, Salinas I (2013) The mucosal immune system of fish: the evolution of tolerating commensals while fighting pathogens. Fish Shellfish Immunol 35: 1729–1739. doi: 10.1016/j.fsi.2013.09.032 PMID: 24099804
60. Rombout JH, Yang G, Kiron V (2014) Adaptive immune responses at mucosal surfaces of teleost fish. Fish Shellfish Immunol 40: 634–643. doi: 10.1016/j.fsi.2014.08.020 PMID: 25150451
61. Uchii K, Okuda N, Minamoto T, Kawabata Z (2013) An emerging infectious pathogen endangers an ancient lineage of common carp by acting synergistically with conspecific exotic strains. Anim Conserv 16: 324–330.
62. Minamoto T, Honjo MN, Yamanaka H, Uchii K, Kawabata Z (2012) Nationwide Cyprinid herpesvirus 3 contamination in natural rivers of Japan. Res Vet Sci 93: 508–514. doi: 10.1016/j.rvsc.2011.06.004 PMID: 21723573
63. Neukirch M, Böttcher K, Bunnajrakul S (1999) Isolation of a virus from koi with altered gills. B Eur Assoc Fish Pat 19: 221–224.
64. Warming S, Costantino N, Court DL, Jenkins NA, Copeland NG (2005) Simple and highly efficient BAC recombineering using galK selection. Nucleic Acids Res 33: e36. PMID: 15731329
65. Simpson JT, Wong K, Jackman SD, Schein JE, Jones SJ, et al. (2009) ABySS: a parallel assembler for short read sequence data. Genome Res 19: 1117–1123. doi: 10.1101/gr.089532.108 PMID: 19251739
66. Li H, Durbin R (2010) Fast and accurate long-read alignment with Burrows-Wheeler transform. Bioinformatics 26: 589–595. doi: 10.1093/bioinformatics/btp698 PMID: 20080505
67. Milne I, Stephen G, Bayer M, Cock PJ, Pritchard L, et al. (2013) Using Tablet for visual exploration of second-generation sequencing data. Brief bioinform 14: 193–202. doi: 10.1093/bib/bbs012 PMID: 22445902
